# *Acanthamoeba castellanii*–Mediated Reduction of Interleukin-1β Secretion and Its Association With Macrophage Autophagy

**DOI:** 10.1155/sci5/3430892

**Published:** 2025-03-12

**Authors:** Rachasak Boonhok, Wilaiwan Senghoi, Suthinee Sangkanu, Chooi Ling Lim, Matsayapan Pudla, Maria de Lourdes Pereira, Polrat Wilairatana, Tooba Mahboob, Md. Atiar Rahman, Pongsak Utaisincharoen, Poonsit Hiransai, Veeranoot Nissapatorn

**Affiliations:** ^1^Department of Medical Technology, School of Allied Health Sciences, and Research Excellence Center for Innovation and Health Products (RECIHP), Walailak University, Nakhon Si Thammarat 80160, Thailand; ^2^Department of Medical Technology, School of Allied Health Sciences, and Center of Excellence Research for Melioidosis and Microorganisms (CERMM), Walailak University, Nakhon Si Thammarat 80160, Thailand; ^3^Department of Pharmacognosy and Pharmaceutical Botany, Faculty of Pharmaceutical Sciences, Prince of Songkla University, Songkhla 90112, Thailand; ^4^Division of Applied Biomedical Science and Biotechnology, School of Health Sciences, International Medical University, Kuala Lumpur 57000, Malaysia; ^5^Department of Oral Microbiology, Faculty of Dentistry, Mahidol University, Bangkok 10400, Thailand; ^6^CICECO-Aveiro Institute of Materials and Department of Medical Sciences, University of Aveiro, Aveiro 3810-193, Portugal; ^7^Department of Clinical Tropical Medicine, Faculty of Tropical Medicine, Mahidol University, Bangkok 10400, Thailand; ^8^Faculty of Pharmaceutical Sciences, UCSI University, Kuala Lumpur 56000, Malaysia; ^9^Department of Biochemistry and Molecular Biology, University of Chittagong, Chittagong 4331, Bangladesh; ^10^Department of Microbiology, Faculty of Science, Mahidol University, Bangkok 10400, Thailand; ^11^Department of Medical Technology, School of Allied Health Sciences, and Center of Excellence in Marijuana, Hemp, and Kratom, Walailak University, Nakhon Si Thammarat 80160, Thailand; ^12^School of Allied Health Sciences, Southeast Asia Water Team (SEA Water Team) and World Union for Herbal Drug Discovery (WUHeDD), Walailak University, Nakhon Si Thammarat 80160, Thailand

**Keywords:** *Acanthamoeba castellanii*, autophagy, human macrophage, inflammasome, interleukin-1β

## Abstract

Noncanonical autophagy including unconventional protein secretion has been extensively studied. Our work focused on a leaderless IL-1β protein secretion from human macrophage in response to *Acanthamoeba castellanii* components, *Acanthamoeba* culture supernatant (CS) and cell lysate (CL), as well as its association with macrophage autophagy. Phorbol 12-myristate 13-acetate (PMA)–induced THP-1 macrophages were treated with *Acanthamoeba* components of pathogenic (ATCC50739) and nonpathogenic (ATCC30010) strains in vitro. The data showed that *Acanthamoeba* treatment resulted in low IL-1β secretion from macrophages. In addition, *Acanthamoeba* CL of both strains was able to upregulate autophagy-related (Atg) protein 8, an autophagy marker, whereas *Acanthamoeba* CS downregulated Atg8 expression. We further manipulated autophagy and found that autophagy induction by starvation diminished IL-1β secretion while autophagy inhibition by 3-methyladenine (3MA) increased IL-1β secretion. Interestingly, in the presence of *Acanthamoeba* components either under starvation or 3MA treatment, IL-1β secretion was significantly reduced. Transcriptional expression of other ATG genes, i.e., ATG6, ATG7, and ATG5, were investigated and showed that their mRNA expression was maintained at the basal level under *A. castellanii* CS or CL treatment. Inflammasome-related genes, NLRP3 and CASPASE1, were upregulated following *A. castellanii* 50739 CS treatment but not in *A. castellanii* 50739 CL-treated condition. However, both conditions were able to increase IL-1β mRNA expression. TEM micrographs revealed that 3MA treatment induced the formation of large vacuoles and accumulation of autophagosome at the edge of THP-1 macrophages. However, the number and size of their structures were declined in the presence of *A. castellanii* 50739 CS with 3MA. Furthermore, immunofluorescence staining demonstrated the association between Atg8/LC3 and IL-1β expression, where downregulation of Atg8 by *A. castellanii* 50739 CS led to the upregulation of IL-1β. Altogether, the data indicate that *Acanthamoeba* can manipulate macrophage autophagy, thereby controlling low IL-1β secretion. The expression of autophagy- and inflammasome-related genes also indicates multiple mechanisms in IL-1β secretion in response to *Acanthamoeba* components. However, further characterization of Atg proteins and investigations into other intracellular pathways or defense mechanisms are needed to fully understand the unconventional secretion of IL-1β in macrophages. This knowledge could eventually lead to the development of innovative therapeutic strategies against *Acanthamoeba* infection by modulating autophagy or macrophage responses.

## 1. Introduction

*Acanthamoeba* species, a free-living parasite, are commonly found in the environment, particularly in soil and water [1, 2]. *Acanthamoeba*, in both its trophozoite and cyst forms, can infect humans through various routes such as the eye, nose, and broken skin, affecting both healthy and immunocompromised individuals. This can lead to a range of mild to severe diseases, including granulomatous amebic encephalitis (GAE), keratitis, and disseminated tissue infections. *Acanthamoeba* keratitis (AK) is the most prevalent eye disease caused by this protozoan parasite, particularly affecting healthy individuals. Recently, its incidence has risen among contact lens wearers [3, 4]. Host immune response to *Acanthamoeba* infection has been extensively studied [5, 6]. Several cytokines, such as interferon-gamma (IFN-γ), interleukin (IL)-4, IL-10, IL-17A, IL-17F, and IL-22, are rapidly produced and secreted in response to *Acanthamoeba* infection [7]. However, the secretion of IL-1β is not well documented, unlike other parasitic infections such as *Toxoplasma gondii* [8] and *Leishmania major* [9].

IL-1 is an early proinflammatory cytokine produced from several cell types including monocytes and macrophages [10]. In humans, the IL-1 family is composed of 11 cytokines, 7 of which have an agonistic activity (IL-1α, IL-1β, IL-18, IL-33, IL-36α, IL-36β, and IL-36γ) to promote inflammation, while the rest possess antagonistic activity (IL-1 receptor antagonist or IL-1Ra, IL-36Ra, IL-37, and IL-38) to exert anti-inflammation [11, 12]. IL-1β plays a crucial role in regulating the inflammatory response. When inflammatory cells encounter pathogen-associated molecular patterns (PAMPs) or damage-associated molecular patterns (DAMPs) that interact with pattern recognition receptors (PRRs) [13], particularly Toll-like receptors (TLRs) [14], pro-IL-1β is rapidly induced. Activation of TLR signaling through the NF-κB pathway results in the production of numerous proinflammatory cytokines, including IL-1β [12]. IL-1β is distinct from other cytokines because it lacks a signal sequence, meaning its secretion bypasses the conventional endoplasmic reticulum (ER)–Golgi pathway. Instead, it is secreted through unconventional protein secretion (UPS) pathways [10]. Numerous proteins lacking a signal peptide that is essential for cell survival, immune surveillance, and tissue organization [15] are also secreted via UPS pathways. However, the secretion mechanisms of these proteins including IL-1β cytokine are not yet completely understood. Autophagy is a degradative pathway that supports cellular viability by removing damaged organelles and protein aggregates, eliminating intracellular microorganisms, and providing the cell with energy and nutrients [16], and the autophagic mechanism also participates in several biogenesis processes including nondegradative role known as secretory autophagy which is one of the forms of UPS [17, 18]. The Type III secretion of UPS is characterized by autophagy-based secretion [15] and a well-established example of this type is IL-1β secretion. This cytokine binds to its specific receptor on both the secreting and neighboring cells, triggering immune cell activation and initiating a strong inflammatory response [19]. Key autophagy-related (Atg) proteins have been reported to be associated with IL-1β secretion after autophagic induction by starvation, i.e., Atg8 and Atg5 [18], as well as Atg7 [20]. Other molecules, such as galectin-8 (a β-galactoside-binding lectin encoded by LGALS8), TRIM16, and SEC22B, have been identified as crucial for the delivery of mature IL-1β to the extracellular environment [21]. Several studies have highlighted the connection between IL-1β secretion and the inflammasome, a multiprotein complex responsible for processing IL-1β prior to its release. The enzymatic cleavage of pro-IL-1β by the protease caspase-1 is initiated when caspase-1 is recruited to multiprotein complexes known as inflammasomes. These inflammasomes consist of adaptor molecules, a cytosolic PRR, and pro-caspase-1. The most well-characterized inflammasome is the NLRP3 inflammasome, which includes the nucleotide-binding oligomerization domain (NOD), leucine-rich repeat (LRR), and pyrin domain-containing protein 3. Pro-caspase-1 binds to ASC, an apoptosis-associated speck-like protein with a caspase recruitment domain, through a homotypic interaction of their CARD domains, resulting in the activation of caspase-1. Following this activation, pro-IL-1β is processed by caspase-1, and the mature IL-1β is rapidly secreted from the cell [10, 22, 23]. This secretion mechanism is regulated by autophagy, which, when induced, leads to the degradation of the inflammasome and a subsequent decrease in IL-1β secretion. This evidence further suggests the involvement of autophagy in regulating the inflammatory response to infection [20].

Our study focuses on the macrophage, an important innate immune cell that provides a protection against many infectious diseases including *Acanthamoeba* infection [24] through several cellular mechanisms such as phagocytic activity, activation of intracellular reactive nitrogen and oxygen species, and secretion of proinflammatory cytokines [25–27], in order to eliminate microbial components or microbes. Macrophage is also a key immune cell that is responsible for IL-1β production in response to pathogens [28, 29]. We examine the secretion of IL-1β from human macrophage and investigate its association with autophagy upon *Acanthamoeba* treatment. A classical autophagy marker, Atg8, including other important autophagy- and inflammasome-related genes in responses to *Acanthamoeba* culture supernatant (CS) and cell lysate (CL) is analyzed. We show that *Acanthamoeba* components are able to control low IL-1β secretion in human macrophage through autophagy regulation and the cytokine secretion likely originates from different protein secretion routes, as suggested by our transcriptional expression data. Additionally, transmission electron microscope (TEM) micrographs show a reduction in the size and number of vacuoles and autophagosomes following *Acanthamoeba* treatment. Furthermore, IFAs of Atg8 and IL-1β also support the notion that *Acanthamoeba* negatively regulates autophagy, which in turn controls IL-1β secretion. This study offers insights into the secretion of the proinflammatory cytokine, IL-1β, following macrophage activation by *Acanthamoeba*, and explores the link between IL-1β secretion and autophagy. These findings could lead to alternative adjunct treatments to modulate immune cell activity and host inflammatory responses during parasitic infections including *Acanthamoeba*.

## 2. Materials and Methods

### 2.1. *Acanthamoeba* Cultivation

*Acanthamoeba castellanii* ATCC50739 (pathogenic strain) and ATCC30010 (nonpathogenic strain) were cultured in PYG medium [2%(w/v) proteose peptone, 0.1% (w/v) yeast extract, 400 μM CaCl_2_, 4 mM MgSO_4_, 2.5 mM Na_2_HPO_4_, 2.5 mM KH_2_PO_4_, 50 μM (NH_4_)2Fe(SO_4_)_2_, 100 mM glucose] in t25 flask at 25°C without shaking [30]. All chemicals were obtained from Sigma-Aldrich (St. Louis, MO, USA). The amoeba trophozoites were cultured in PYG media, with fresh media being replaced every 2-3 days until the *Acanthamoeba* were harvested. To prepare *Acanthamoeba* trophozoite for parasite sample collection, 1 × 10^5^ cells were seeded in t25 flask for 5 days. The *Acanthamoeba* CS was first collected and centrifuged at 4°C, 3000 rpm, 5 min to remove parasite pellet. Later, the trophozoite was flushed with PYG and centrifuged at 4°C, 3000 rpm, 5 min to collect a parasite pellet. The pellet was saved in 1.5-mL Eppendorf tube and then subjected to three freeze–thaw cycles by freezing at −80°C for 30 min and thawing at room temperature (RT) for 15 min. Subsequently, the samples were sonicated at 40% amplitude with 10-s pulses followed by 15-s intervals for a total of 2 min. The whole CL was kept at −20°C until use.

### 2.2. Human Monocyte THP-1 Cultivation and Differentiation to Macrophages

Human THP-1 acute monocytic leukemia (THP-1 AML or THP-1) cell line was cultured with RPMI 1640 (Invitrogen, USA) supplemented with 10% heat-inactivated fetal bovine serum (InvivoGen, USA) (known as RPMI1640-10) and maintained in a humidified 5% CO_2_ incubator, 37°C. To differentiate THP-1 monocytic cells into macrophages, the cells were treated with 5 ng/mL phorbol 12-myristate 13-acetate (PMA) (Sigma, P8139) for 48 h [31].

### 2.3. Human IL-1β Detection by ELISA

To detect IL-1β, the macrophage was treated with the *Acanthamoeba* CS and CL of both strains, ATCC50739 and ATCC30010. Lipopolysaccharide (LPS) (InvivoGen, CA, USA) of 1 μg/mL was used as a positive control to stimulate IL-1β secretion from macrophages. At 24 h post-treatment, the CS was harvested. Then, IL-1β secretion analysis and cell viability were analyzed. The human IL-1β was determined by ELISA (ELISA MAX Deluxe Sets, Cat. No, 437004, Biolegend, San Diego, USA). Detection was carried out following the manufacturer's instructions, with each sample tested in triplicate. Additionally, an MTT (3-(4,5-dimethylthiazol-2-yl)-2,5-diphenyltetrazolium bromide) assay (Sigma, St. Louis, USA) was conducted to evaluate cell viability post-treatment.

To induce autophagy by starvation, an Eagle's balanced salt solution medium or EBSS (137 mM sodium chloride, 5 mM potassium chloride, 1 mM magnesium sulfate, 1 mM calcium chloride, 10 mM glucose, and 0.004 mM phenol red, often supplemented with 4.2 mM sodium bicarbonate) (Invitrogen, San Diego, CA, USA) was used and different regimens for THP-1 macrophage treatment were performed with the following conditions: (1) 4-h starvation followed by *Acanthamoeba* treatment; (2) 4-h starvation with *Acanthamoeba* followed by *Acanthamoeba* treatment; (3) 2-h *Acanthamoeba* pretreatment followed by 4-h starvation and *Acanthamoeba* treatment; (4) 4-h starvation with *Acanthamoeba* and no subsequent *Acanthamoeba* treatment. Inhibition of autophagy by 3MA (Sigma-Aldrich, St. Louis, MO, USA), the autophagy inhibitor at 1 mM was coincubated with the *Acanthamoeba* along the treatment. The THP-1 macrophage CS was harvested at 24 h post-treatment, and IL-1β was detected by ELISA. LPS treatment, 4-h starvation, 3MA treatment alone, and 3MA with LPS treatment were also included in this assay.

### 2.4. Cytotoxicity Test by MTT Assay

An MTT assay was performed to determine the cell viability. MTT powder (Sigma, St. Louis, MO) was prepared in PBS as a stock at 5 mg/mL and sterilized using a 0.2-μm filter. After the treatment, THP-1 macrophages were incubated with 100 μL of MTT solution at a final concentration of 0.5 mg/mL in culture medium, 37°C with 5% CO_2_ for 2 h in the dark. The viable cells metabolize the MTT into the dark blue formazan crystals. Then, the reagent was gently discarded, the formazan crystals were dissolved with 200 μL of dimethyl sulfoxide (DMSO), and the plate was incubated overnight at RT to completely dissolve the formazan crystals. The optical density (OD) was determined at 550 and 630 nm by a microplate reader. The percentage of cell viability was calculated as described below. OD_550_ of all conditions were subtracted with OD_630_, the average OD_550–630_ from triplicate was determined, the OD_control_ (untreated condition) was set to 100% cell viability, and then, the % cell viability of OD_samples_ was calculated accordingly [32].

### 2.5. Preparation of Total RNA and cDNA Synthesis

THP-1 macrophages in 24-well plates obtained from different conditions were harvested at 4, 8, and 24 h post-treatment. The medium was discarded; then, the cells were lysed with 500 μL of TRI reagent (Molecular Research Center, Cincinnati, USA) to preserve their RNA. Total RNA extraction was carried out using an RNA extraction kit (Vivantis Technologies, Selangor, Malaysia). Subsequently, 100 ng of mRNA was converted to cDNA using the Viva cDNA synthesis kit (Vivantis Technologies, Selangor, Malaysia) according to the manufacturer's instructions. The cDNA was then stored at −20°C until use.

### 2.6. Analysis of Gene Expression by Quantitative PCR

All qPCR primers were listed as follows: ATG6 [33], F 5′-GAACCGCAAGATAGTGGC-3′ and R 5′-CAGAGCATGGAGCAGCAA-3′; ATG7 [34], F 5′-AACCTCTCTTGGGCTTGTGC-3′ and R 5′-GGCTGACGGGAAGGACATT-3′; ATG5 [35], F 5′-GCAAGCCAGACAGGAAAAAG-3′ and R 5′-GACCTTCAGTGGTCCGGTAA-3′; NLRP3 [36], F 5′-GATCTTCGCTGCGATCAACA-3′ and R 5′-GGGATTCGAAACACGTGCATTA-3′; CASPASE 1 [36], F 5′-GCCTGTTCCTGTGATGTGGAG-3′ and R 5′-TGCCCACAGACATTCATACAGTTTC-3′; IL-1β (OriGene Technologies, Rockville, Maryland), F 5′-CCACAGACCTTCCAGGAGAATG-3′ and 5′-GTGCAGTTCAGTGATCGTACAGG-3′; and GAPDH [37], F 5′-ACACCCACTCCTCCACCTTT-3′ and R 5′-TAGCCAAATTCGTTGTCATACC-3′.

The qPCR was prepared using the iTaq Universal SYBR Green Supermix Kit from Bio-Rad (Bio-Rad Laboratories, Hercules, USA) by the manufacturer's instructions. The GAPDH was used as a reference gene. The qPCR preparation and qPCR cycling by StepOnePlus Real-time PCR systems (Applied Biosystems, Waltham, USA) were conducted according to the protocol described by Boonhok, et al. in 2022 [38]. In brief, the thermal cycler software was configured as follows: holding stage at 95°C for 30 s, cycling stage for 40 cycles at 95°C for 15 s, 60°C for 60 s, and melting curve stage at 95°C for 15 s, 60°C for 60 s, and 95°C for 15 s with a temperature increase of 0.3°C. The delta-delta Ct (ΔΔCt) and relative mRNA expression were calculated using the following formula: ΔΔCt = [(Ct of treated sample GOI − Ct of treated sample housekeeper) − (Ct of untreated control GOI − Ct of untreated control housekeeper)], where GOI represents the gene of interest. The relative mRNA expression was then calculated as 2 to the power of (minus *X*) or 2^−*X*^, where *X* is ΔΔCt. The results are interpreted as follows: A value above 1 indicates increased expression, a value below 1 indicates decreased expression, and a value of 1 indicates constant expression.

### 2.7. SDS–PAGE and Immunoblotting

The THP-1 macrophages were lysed using a lysis buffer which is composed of 20 mM Tris, 100 mM NaCl, and 1% Nonidet P-40. The lysates were then separated by SDS–PAGE on an 8% or 15% (w/v) polyacrylamide gel, followed by the transfer of proteins onto a nitrocellulose membrane (Pall). The membranes were blocked with a 5% (v/v) blocking solution (Roche Diagnostics) for 1 h, and then, primary antibodies were added against IL-1β (Cell Signaling Technology (CST), Cat#12242, mouse mAb, Danvers, Massachusetts, USA) (1:1000), Atg8 (Novus Biologicals, Cat#NB100-2220, rabbit pAb, Littleton, Colorado, USA) (1:2000), and actin (mouse mAb, Cat# MAB1501R, Merck Millipore, Burlington, VT, USA) (1:20,000) as an internal control at 4°C overnight. Following 4 washes with 0.1% PBST, the membranes were incubated with the appropriate HRP-conjugated secondary antibodies (anti-mouse and anti-rabbit 1:5000) (Pierce, Thermo Fisher Scientific, Waltham, MA, USA) for 1 h at RT. After another 4 washes with 0.1% PBST, the membranes were incubated with a chemiluminescence substrate (Roche Diagnostics). The proteins were subsequently detected using the enhanced chemiluminescence method [39].

### 2.8. Ultrastructural Analysis by TEM

THP-1 monocytes were cultured in a 6-well plate at 5 × 10^5^ cells/well. After 48-h PMA treatment, cells were treated with *Acanthamoeba castellanii* ATCC50739 CS with or without 3MA. THP-1 macrophage treated with 3MA alone and THP-1-untreated condition were used as controls. After 24-h treatment, cells were washed with PBS 1 time, detached by a cell scraper, transferred to a conical tube, and fixed with 2.5% glutaraldehyde for 2 h. Then, the cells were washed twice with PBS and finally kept in the PBS. The samples were shipped to the Office of Scientific Instrument and Testing (OSIT), Prince of Songkla University, Thailand, for TEM. In brief, the sample was cut into 0.1–0.5 mm^3^, fixed with 2.5% glutaraldehyde for 2 h and washed 3 times with PBS, and then fixed with 1% osmium for 2 h and washed with distilled water (DW) 3 times. En bloc-staining with 2% uranyl was then performed for 20 min and dehydrated with a series of ethanol (70%, 80%, 90%, and 100%). Next, the sample was infiltrated with the following reagents: propylene oxide for 15 min, propylene oxide:epoxy resin (1:1) for 2 h, propylene oxide:epoxy resin (1:2) for 2 h, and pure epoxy for 3 h. Embedding was then performed into a capsule, dried at 70°C–80°C for 30 min, and completely filled the capsule with the pure epoxy resin. The sample was then left in the incubator to enhance polymerization, and sectioning of the sample was performed by ultramicrotome.

### 2.9. IFA

THP-1 macrophages of 3 × 10^5^ cells/well were prepared in a 24-well plate containing a circular coverslip. Then, the cells were pulsed with *Acanthamoeba* CS or CL of *A. castellanii* ATCC50739. After 24 h post-treatment, the IFA was performed by fixing with 4% paraformaldehyde/PBS for 10 min, washing with PBS, permeabilizing cells with 0.1%Triton X-100/PBS for 10 min, washing with PBS, and blocking of nonspecific binding with 3%BSA/PBS at RT for 30 min. The primary antibody, rabbit anti-LC3 (Novus Biologicals, Cat#NB100-2220, Centennial, CO, USA) (dilution 1:100), was used for cell staining at RT for 1 h. After 3 times washing with PBS, 10 min each, the cells were stained with the secondary antibody, donkey anti-rabbit IgG-conjugated A555 (Biolegend, Cat# BIOL-406412, San Diego, CA, USA) (dilution 1:250) at RT in the dark for 1 h, and then washed 4 times with PBS. The cells were then stained with rabbit anti–IL-1β-conjugated A488 (Novus Biologicals, Cat# NBP1-19775AF488, Centennial, CO, USA) (dilution 1:200) at RT for 45 min. After washing, the coverslips were placed upside down onto the glass slide containing 1 drop of DAPI (SouthernBiotech, Cat#0100-20, Birmingham, AL, USA). The slide samples were sealed and analyzed by Leica SP5 II laser scanning confocal microscope (Wetzlar, Germany). The number of LC3 puncta-containing cells was quantified by examining at least 100 cells per condition from two independent experiments. Only puncta with a size of 0.25 μm or larger were counted [40].

### 2.10. Statistical Data Analysis

All data were collected from three independent experiments. The results were combined to calculate the mean ± standard deviation (SD) or standard error of the mean (SEM), including real-time PCR data (ddCt values). Data analysis was performed using a two-tailed unpaired Student's *t*-test with Prism 5 software. A *p* value of < 0.05 was considered statistically significant.

## 3. Results

### 3.1. IL-1β Secretion and Autophagic Response in THP-1 Macrophage to *Acanthamoeba castellanii* Treatment

The optimal conditions for PMA-induced THP-1 differentiation and *Acanthamoeba* CS and CL preparation were examined. PMA-induced THP-1 differentiation for 48 h was used throughout the study. Day 3 of *Acanthamoeba* culture, which was in a log phase of *Acanthamoeba* growth, was harvested. The *Acanthamoeba* CS was used for the THP-1 macrophage treatment at 2% (v/v), while *Acanthamoeba* CL culture was used at 10% (v/v) (8 μg total protein) as these conditions were able to induce IL-1β secretion with no cytotoxic effect (Figure S1). *Acanthamoeba* culture medium, PYG, alone was also tested for its ability to induce IL-1β secretion in THP-1 macrophage. The results demonstrated that IL-1β secretion was not increased even at 10%, the highest percentage of PYG (Figure S1).

**Figure 1:**
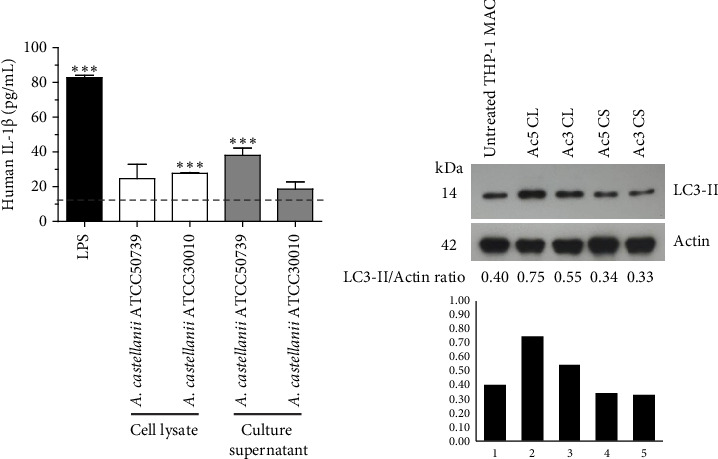
IL-1β secretion and autophagic response in THP-1 macrophages treated with *Acanthamoeba castellanii.* The THP-1 macrophages were treated with *A. castellanii* ATCC50739 (Ac5) or 30010 (Ac3) cell lysate (CL) or culture supernatant (CS) for 24 h. (a) The culture supernatant of THP-1 macrophage culture was collected for IL-1β detection by ELISA. LPS-treated condition was included as a positive control for IL-1β secretion. The dotted line represented the basal level of IL-1β secretion (untreated cells). Data were obtained from three independent experiments. Bar graphs show mean ± SD. ⁣^∗∗∗^*p* < 0.001. (b) The THP-1 macrophages were harvested for Western blot analysis of Atg8/LC3. Actin was included as an internal control. The LC3-II/Actin ratio was analyzed and represented as a histogram.

The basal level of IL-1β secretion by THP-1 macrophages (untreated condition) was approximately 16.40 pg/mL, while in LPS-treated condition, a positive control, IL-1β secretion was significantly increased. In *Acanthamoeba* ATCC50739 CS- and ATCC30010 CL-treated THP-1 macrophages, IL-1β secretion was significantly increased, whereas under *Acanthamoeba* ATCC50739 CL- and ATCC30010 CS-treated THP-1 macrophages, the IL-1β secretion was slightly changed (Figure 1(a)). Upon autophagy induction, the cytosolic form LC3-I is converted to its mature lipidated form, LC3-II, which associates with intracellular membranes to facilitate autophagosome formation. The expression of LC3-II, a key autophagic marker, was analyzed following *Acanthamoeba* treatment using Western blotting. Our data showed that *Acanthamoeba* CL, particularly from ATCC50739, induced autophagy, while *Acanthamoeba* CS from both ATCC30010 and ATCC50739 led to a modest reduction in LC3-II expression, suggesting autophagy downregulation. The effects of *Acanthamoeba* components on autophagy were inversely correlated with IL-1β secretion. However, in the ATCC30010 CS-treated condition, IL-1β secretion did not increase despite autophagy impairment, indicating a potential strain-specific regulation of IL-1β production independent of autophagy suppression (Figure 1(b)).

### 3.2. Manipulation of Autophagy in THP-1 Macrophage and Analysis of IL-1β Secretion

**Figure 2:**
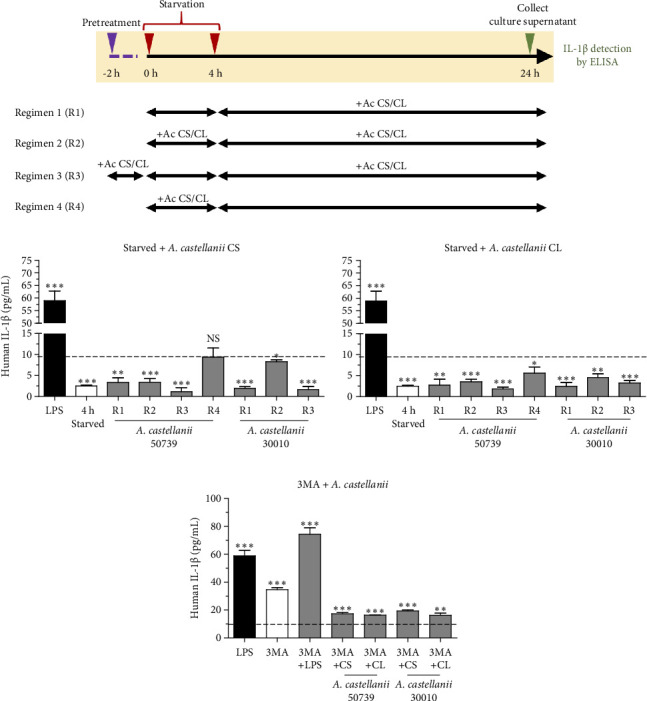
Manipulation of autophagy in THP-1 macrophages and IL-1β secretion. (a) Different regimens for THP-1 macrophage starvation. (b) Autophagy induction by starvation, THP-1 macrophages were starved for 4 h in the presence of *Acanthamoeba* culture supernatant (CS) (left) or cell lysate (CL) (right) of *A. castellanii* 50739 and 30010. (c) Inhibition of autophagy by 3MA, the THP-1 macrophages were treated with the autophagy inhibitor at 1 mM in the presence or absence of *Acanthamoeba* CS or CL. THP-1 macrophage culture supernatant was harvested at 24 h post-treatment and IL-1β was detected by ELISA. LPS treatment, 4-h starvation, 3MA treatment alone, and 3MA with LPS treatment were included as controls. Dotted line represented a basal level of IL-1β secretion in THP-1 macrophages. Data are obtained from three independent experiments and represented as mean ± SD. NS, not significant; ⁣^∗^*p* < 0.05; ⁣^∗∗^*p* < 0.01; ⁣^∗∗∗^*p* < 0.001.

To test whether macrophage autophagy is involved in IL-1β secretion upon *Acanthamoeba* activation, we first induced autophagy in THP-1 macrophage through starvation or nutrient depletion, a classical autophagy induction in vitro, and different starvation regimens as shown in Figure 2(a) were set up to examine the IL-1β secretion in the presence of *Acanthamoeba* components, i.e., *Acanthamoeba* CS or CL of ATCC50739 or 30010 strain. In brief, Regimen 1 (R1) was a 4-h starvation with EBSS medium and then replaced with fresh complete/full RPMI-10 plus *Acanthamoeba* CS or CL; R2 was similar to R1, and however, the difference was the *Acanthamoeba* CS or CL was coincubated during 4-h starvation; R3 was also similar to R1 and called the pretreatment regimen as the THP-1 macrophages were pretreated with *Acanthamoeba* CS or CL for 2 h before starvation; R4 was similar to R2, but after 4-h starvation, the *Acanthamoeba* CS or CL was not included for the treatment. In addition, starvation alone (EBSS medium) was included as a control in which the THP-1 macrophages were starved for 4 h and then replaced with full RPMI-10. The LPS-treated condition was included as a positive control for IL-1β secretion, while the untreated condition was included as a control to indicate a basal level of IL-1β secretion. On the other hand, inhibition of autophagy by 3-methyladenine (3MA), a classical autophagy inhibitor, was also conducted. After 24-h treatments, the secreted IL-1β was analyzed. Manipulation of autophagy was confirmed by Western blot analysis of LC3/Atg8 (Figure S2A).

Our results demonstrated that the basal level of IL-1β secretion in THP-1 macrophage was approximately 9.1 pg/mL represented by the dotted line. LPS treatment significantly induced IL-1β secretion, 58.90 pg/mL, whereas 4-h starvation alone significantly reduced IL-1β secretion, 2.48 pg/mL (Figure 2(b)). Interestingly, in the presence of *Acanthamoeba* CS or CL from either ATCC50739 or 30010, IL-1β secretion was reduced compared to the untreated condition, as demonstrated in Figure 2(b) (left and right). Noteworthy, the R4 regimen demonstrated that in the absence of *Acanthamoeba* ATCC50739 CS or CL after 4-h starvation, the IL-1β secretion was able to recover and close to the basal level. Upon autophagy inhibition by 3MA, the IL-1β secretion was significantly increased compared to untreated control, 34.59 pg/mL, and even higher in the presence of 3MA plus LPS, 74.34 pg/mL (Figure 2(c)). However, in the presence of 3MA together with *Acanthamoeba* CS or CL of ATCC50739 or 30010, the IL-1β secretion was significantly reduced compared to the 3MA treated alone but remained significantly higher than that of the untreated control. Additionally, pro-IL-1β protein expression in the cell pellet from the tested conditions was analyzed using Western blotting to determine whether the low IL-1β secretion in *Acanthamoeba* treatment was due to defective enzymatic cleavage of pro-IL-1β protein or impaired transport of mature IL-1β out of the cell. Unfortunately, pro-IL-1β protein was detected only in the conditions treated with LPS, 3MA, and 3MA combined with LPS, while the mature IL-1β protein was absent in the cell pellet (Figure S2B). Furthermore, cell viability testing following *Acanthamoeba* treatment was conducted using the MTT assay, and the selected conditions did not affect the viability of THP-1 macrophages (Figure S3). The data indicate a crucial role of autophagy in controlling IL-1β secretion; however, in the presence of *Acanthamoeba* components, IL-1β is regulated and secreted at a low level.

### 3.3. Transcriptional Expression of Atg Genes in THP-1 Macrophage in Response to *Acanthamoeba castellanii*

**Figure 3:**
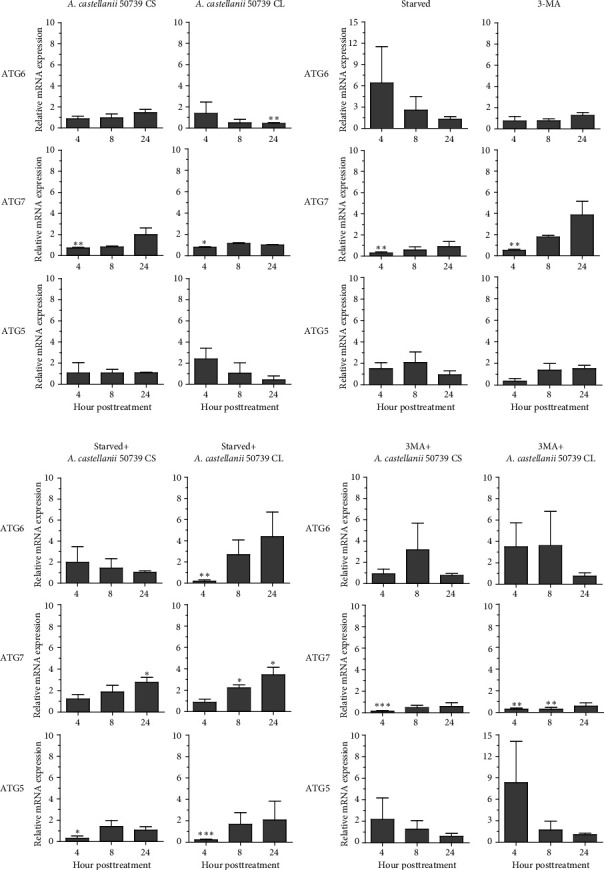
Transcriptional expression of autophagy-related genes in THP-1 macrophages. THP-1 macrophages were treated with *A. castellanii* ATCC50739 culture supernatant (CS) or cell lysate (CL) alone or treatment under starvation and 3MA. The cells were harvested at 4, 8, and 24 h post-treatment, and the mRNA expression of ATG6, ATG7, and ATG5 was analyzed by qPCR. (a) *Acanthamoeba* treatment under full condition, RPMI-10. (b) 4-h starvation and 24-h 3MA treatment alone. (c) 4-h starvation with *Acanthamoeba* treatment. (d) 3MA treatment with *Acanthamoeba* treatment. Bar graphs represent mean ± SEM. ⁣^∗^*p* < 0.05; ⁣^∗∗^*p* < 0.01; ⁣^∗∗∗^*p* < 0.001.

In addition to Atg8 protein, we investigated the mRNA expression of other key Atg genes, i.e., ATG6, ATG7, and ATG5 in THP-1 macrophages in response to *A. castellanii* ATCC50739 CS and CL. The *Acanthamoeba*-treated alone and manipulation of autophagy alone or in combination with *Acanthamoeba* components were included. The results showed that under full condition, *A. castellanii* CS and CL slightly affected the mRNA expression of ATG6, ATG7, and ATG5 and their expression was maintained at the basal level along the treatment (Figure 3(a)). Upon autophagy manipulation, ATG6 mRNA was upregulated at 4 h post-treatment, whereas ATG7 and ATG5 were slightly changed and remained at the basal level in starvation alone. In 3MA-treated alone, all tested ATG genes were unchanged, while ATG7 was slightly increased at later time points (Figure 3(b)). In starvation with *A. castellanii* CS, the mRNA expression of ATG6 and ATG5 was remained at the basal level while ATG7 was gradually increased, whereas in starvation with *A. castellanii* CL, the mRNA expression of ATG6, ATG7, and ATG5 showed a similar expression pattern, and all were progressively increased along the treatment (Figure 3(c)). On the other hand, in 3MA treatment with *A. castellanii* CS or CL, a similar mRNA expression pattern of the tested ATG genes was observed. At early time points post-treatment, the expression was slightly changed; however, at 24 h post-treatment, the expression of those genes was declined to the basal level (Figure 3(d)). The transcriptional expression data indicate the effect of *Acanthamoeba* components in maintaining the key Atg genes at the basal level in macrophages which subsequently control IL-1β secretion.

### 3.4. Transcriptional Expression of Inflammasome-Related Genes in THP-1 Macrophage in Response to *Acanthamoeba castellanii*

**Figure 4:**
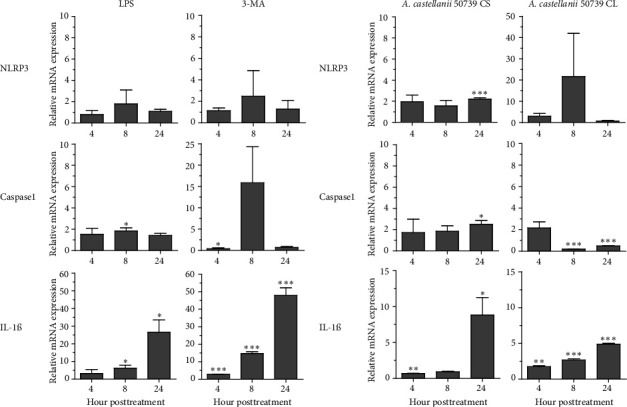
Transcriptional expression of inflammasome-related genes in THP-1 macrophages. (a) THP-1 macrophages were treated with 24-h LPS and 3MA alone. (b) THP-1 macrophages were treated with *A. castellanii* ATCC50739 culture supernatant (CS) or cell lysate (CL). The cells were harvested at 4, 8, and 24 h post-treatment, and the mRNA expression of NLRP3, CASPASE1, and IL-1β was analyzed by qPCR. Bar graphs represent mean ± SEM. ⁣^∗^*p* < 0.05; ⁣^∗∗^*p* < 0.01; ⁣^∗∗∗^*p* < 0.001.

Regarding the role of inflammasome in IL-1β secretion, thus, some of the key inflammasome-related genes, i.e., NLRP3 and CASPASE1 including IL-1β, were investigated for their expression in response to *A. castellanii* ATCC50739 (Figure 4). LPS and 3MA treatment alone were included as controls as they effectively induced IL-1β secretion. LPS slightly induced the upregulation of NLRP3 and CASPASE1. However, IL-1β mRNA expression at 8 and 24 h post-treatment was significantly increased. In 3MA treatment, a similar mRNA expression pattern was observed and the IL-1β expression was significantly increased in particular at 24 h post-treatment (Figure 4(a)). Upon *A. castellanii* treatment, *A. castellanii* CS faintly affected their mRNA expression at early time points. However, at 24 h post-treatment, their mRNA expression was significantly increased. This was slightly different from *A. castellanii* CL treatment. In *A. castellanii* CL-treated condition, NLRP3 expression was fluctuated, increased at 8 h post-treatment, and later declined to the basal level, while CASPASE1 expression was downregulated at 8 and 24 h post-treatment. However, the IL-1β mRNA expression was significantly increased along the treatment but still lower than *A. castellanii* CS treatment (Figure 4(b)). The transcriptional expression data reveal that treatment with *Acanthamoeba* CS, which has been shown to attenuate autophagy, upregulates the expression of inflammasome-related genes, including IL-1β. However, treatment with *Acanthamoeba* CL, which also induces IL-1β expression, may involve other IL-1β secretion pathways independent of NLRP3 and caspase-1.

### 3.5. Ultrastructural Analysis of THP-1 Macrophage in Response to *Acanthamoeba castellanii*

**Figure 5:**
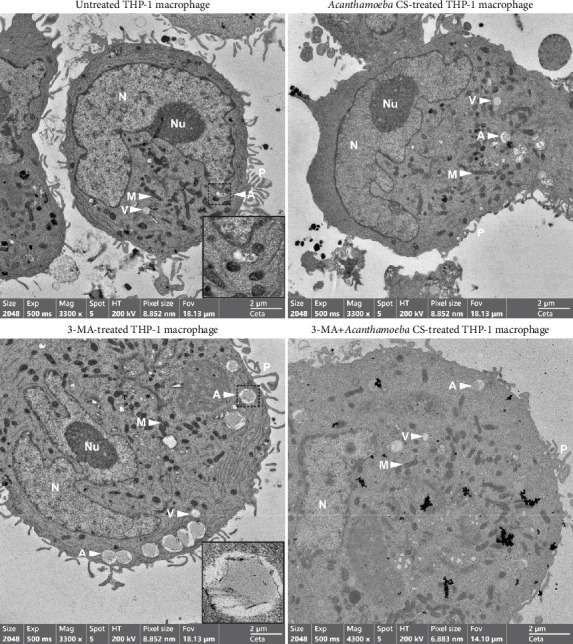
Ultrastructural analysis of THP-1 macrophages by TEM. THP-1 macrophages were treated with *A. castellanii* ATCC50739 culture supernatant (CS) with or without 3MA. Untreated condition was included as a control. Structural features identified include P, pseudopodia; N, nucleus; Nu, nucleolus; L, lysosomal granules; ER, endoplasmic reticulum; M, mitochondria; V, vesicle/vacuole; A, autophagosome. The insets highlight autophagosomes of different sizes. Bar: 2 μm.

Ultrastructural changes as well as the expression and localization of proteins of interest in THP-1 macrophages in response to *Acanthamoeba* ATCC50739 treatment were investigated. TEM micrographs revealed the typical hallmarks of PMA-induced THP-1 macrophages, including spread cell morphology with cell adhesion, the presence of pseudopodia, an irregularly shaped nucleus, and increased granularity. Additionally, intracellular compartments such as mitochondria, ER, vesicles/vacuoles, and autophagosomes were clearly observed (Figure 5 Top-Left). To enhance visualization, an inset at the bottom-right corner highlights the autophagosome, a characteristic double-membrane structure. Upon *A. castellanii* CS treatment, the reduction of pseudopodia was observed. However, other cell structures were similar to the untreated control (Figure 5 Top-Right). In 3MA-treated THP-1 macrophages, the ultrastructure exhibited slight differences compared to the control. Large autophagosomes were observed, with an increased number accumulating at the cell edge. Large vacuoles were also evident. However, the morphology of mitochondria, ER, nucleus, and pseudopodia remained similar to the untreated control. To provide a clearer view of the autophagosome structure, an inset was included in Figure 5 (Bottom-Left). Interestingly, in the presence of 3MA with *A. castellanii* CS, the number of autophagosomes was reduced to the basal level and other structures were similar to the *A. castellanii* CS-treated alone (Figure 5 Bottom-Right). The TEM micrographs reveal the key ultrastructures of human macrophages and their physiological changes upon autophagy inhibition by 3MA, which possibly supports the transport of IL-1β out of the cell as well as the ability of *Acanthamoeba* CS to control the changes that mediate low IL-1β secretion.

### 3.6. IL-1β and LC3/Atg8 Expression and Localization in THP-1 Macrophage in Response to *Acanthamoeba castellanii*

**Figure 6:**
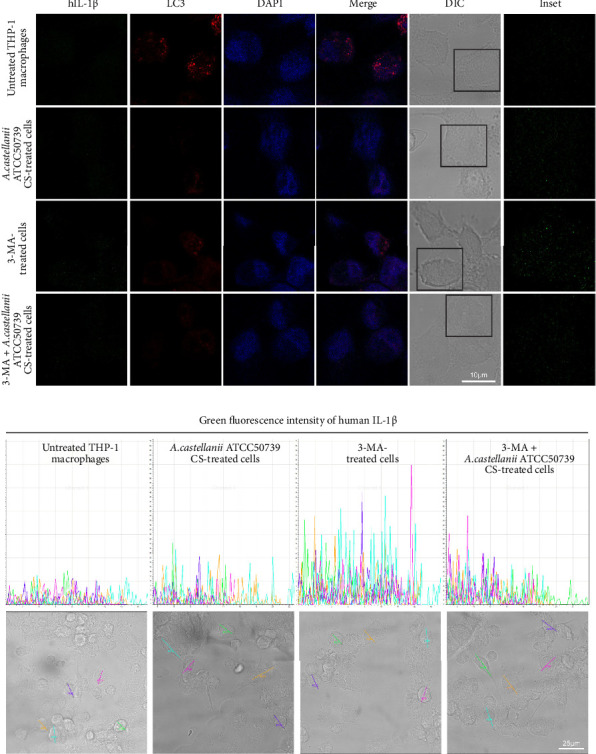
Representative images of human IL-1β and LC3 protein expression and localization in THP-1 macrophages. (a) The cells were pulsed with *A. castellanii* ATCC50739 culture supernatant (CS) or 3MA alone or in combination of the *Acanthamoeba* CS and 3MA. Untreated condition was included as a control. The immunofluorescence assay was performed to observe the expression of human IL-1β (Green) and human LC3 (Red). DAPI (Blue) and DIC were included to show cell nucleus and cell boundary, respectively. Insets represent the expression of hIL-1β. Bar 10 μm. (b) Histograms showing green fluorescence intensity of human IL-1β in THP-1 macrophages. The cells were obtained from different conditions. Five cells per condition were selected and demonstrated the IL-1β protein expression through a green fluorescence signal. *X*-axis represents the maximum ROI length in μm. *Y*-axis represents the green fluorescence intensity (0–70). Bar: 25 μm.

IFA was performed to investigate the IL-1β and LC3/Atg8 protein expression and their localization in THP-1 macrophage (Figure 6(a)). Due to the very low expression of IL-1β which was below the detection limit of this technique, thus, it was unable to clearly demonstrate this protein. However, the image analysis was performed to investigate the IL-1β expression through the detection of green fluorescence intensity. The region of interest (ROI) was drawn across the cells, and five representative ROIs per condition were demonstrated (Figure 6(b)). On the other hand, LC3 protein expression and localization were examined. In untreated control, the LC3 expression was observed and showed its localization in the THP-1 cytosol (Figure 6(a)). The average number of LC3 puncta per cell was 4.76 (Figure S4). However, upon *A. castellanii* ATCC50739 CS treatment, the reduction of LC3 was demonstrated and the average number of LC3 puncta per cell was 0.25. 3MA-treated THP-1 macrophage was included as a control for autophagy inhibition and IL-1β secretion, and its LC3 expression was reduced compared to untreated control and the average number of LC3 puncta per cell was 0.88. Moreover, in the presence of 3MA together with *A. castellanii* ATCC50739 CS, as expected, the LC3 expression was also decreased similar to *A. castellanii* ATCC50739 CS or 3MA treatment alone and the average number of LC3 puncta per cell was 0.54. Interestingly, the data from image analysis of IL-1β expression (green fluorescence intensity) by drawing the ROIs also showed its association with LC3 expression (Figure 6(b)). In untreated THP-1 macrophages, LC3 expression was clearly seen, while the IL-1β expression was very low. In the presence of 3MA alone, the LC3 expression was reduced, whereas the IL-1β expression was increased. Upon *A. castellanii* ATCC50739 CS treatment alone or the combination with 3MA, the LC3 expression was low similar to 3MA-treated alone, while the IL-1β was slightly increased. These data demonstrate the link between autophagy protein markers and IL-1β expression, as well as the effect of *Acanthamoeba* CS in downregulating autophagy and upregulating IL-1β.

## 4. Discussion

Several cytokines, for example, IFN-γ, IL-4, IL-10, IL-17A, IL-17F, and IL-22, were reported to rapidly produce and secrete in response to the *Acanthamoeba* infection [7] but not IL-1β cytokine. Our study focused on IL-1β secretion from human macrophage in response to *Acanthamoeba castellanii*. IL-1β agonistic molecule is basically synthesized as a precursor of 31 kDa and further processed by proteases to its mature secreted form of 17 kDa. In our research, *Acanthamoeba* components, i.e., the CS and whole CL of *A*. *castellanii* of pathogenic strain, ATCC50739, and nonpathogenic strain, ATCC30010, were used as a PAMP to activate THP-1 macrophages and IL-1β secretion was investigated. Our data revealed that *Acanthamoeba* CS and CL of both strains were able to induce IL-1β secretion from the human macrophage which was similar to other microbial infections, for example, *Leishmania* spp. [41], *Toxoplasma gondii* [42], and *Mycobacterium* spp. [25]. However, *Acanthamoeba* CS of the pathogenic strain had higher capacity of inducing IL-1β secretion compared to the nonpathogenic strain. This may indicate a difference of protein profile in the CS or virulence factor between these 2 strains and requires additional research. However, these data were different from mycobacterial infection in which the IL-1 secretion from macrophage was varied independently of pathogenic or nonpathogenic strains of the *Mycobacteria* [25]. Moreover, due to the low IL-1β secretion upon *Acanthamoeba* treatment, we further investigated the two forms of IL-1β, pro-IL-1β and mature IL-1β, in the cell pellet after the *Acanthamoeba* activation. Our Western blot analysis data of IL-1β forms showed that under *Acanthamoeba*-treated conditions, those IL-1β expression was below the detection limit. However, in conditions of LPS, 3MA, and 3MA combined with LPS treatment, the band of pro-IL-1β protein but not mature IL-1β was observed. This indicates a normal enzymatic cleavage of pro-IL-1β and transportation of mature IL-1β under these conditions, and further investigations are needed for *Acanthamoeba*-treated conditions to understand IL-1β processing and secretion.

Regarding an UPS, a large number of proteins that lacks a signal peptide are secreted without entering a conventional protein secretion pathway and these proteins play roles in various cellular functions, including cell survival, immune surveillance, and tissue organization [15]. The UPS pathways have been reported to be associated with the autophagy pathway [18, 43]. Autophagy is an intracellular degradative and disposal pathway supporting cell survival by removing damaged organelles, protein aggregates, intracellular microorganisms, and supplying nutrients to the cell [16]. The autophagic mechanism also participates in several biogenesis processes including nondegradative role known as secretory autophagy [17, 18]. The Type III UPS is characterized by autophagy-based secretion [15] or known as an ATG gene-dependent secretion [43]. A well-known model for this type of UPS is IL-1β secretion. In canonical autophagy, ATG8 lipidation is a hallmark where Atg proteins participate in autophagosome formation, fusion with lysosomes, and cargo selection. ATG7 is an essential core ATG protein that promotes the key stages of classical degradative autophagy through ATG8 lipidation [44]. Furthermore, ATG7 has important functions in innate immunity, such as LC3-associated phagocytosis, UPS, receptor recycling, exocytosis of secretory granules, and the regulation of cell cycle arrest and apoptosis [45, 46]. The study by Ying, et al. in 2021 revealed that Astragaloside IV induced autophagy by inhibiting the TLR4/NF-κB signaling pathway, leading to a reduction in the release of inflammatory cytokines, such as IL-1β, from mouse macrophages. Additionally, its crucial role in influencing Atg genes, including ATG7, ATG5, and ATG6, was demonstrated [20]. The Atg12-Atg5-Atg16 complex is essential for promoting efficient Atg8 lipidation [47]. The study by Nicolas Dupont et al. in 2011 also discovered that the stimulation of autophagy simultaneously with inflammasome agonists, for example, nigericin, alum, or silica significantly enhanced IL-1β secretion in an Atg5-dependent manner. Additionally, intracellular IL-1β was colocalized with Atg8/LC3 puncta, demonstrating the intersection between the autophagy process and IL-1β secretion [48]. Atg6/Beclin-1 has a slightly different role, being involved in the early phase of autophagosome formation, known as nucleation, which relies on Beclin 1–Vps34–Vps15 core complexes. It also participates in autophagosome maturation, requiring several proteins including Atg8, lysosomal membrane proteins, and the GTP-binding protein RAB7 [49]. However, there are several molecules, for example, galectin-8, a β-galactoside-binding lectin encoded by LGALS8, TRIM16, SEC22B, etc., that have been mentioned to be crucial for delivering mature IL-1β to extracellular environment [21].

In our work, a classical autophagic marker, Atg8/LC3-II protein, including a transcriptional expression of ATG5, ATG6, and ATG7 was analyzed. Our results demonstrated that the *Acanthamoeba* CL of both strains was able to induce autophagy in human macrophage through Atg8/LC3-II Western blot analysis, whereas *Acanthamoeba* CS treatment slightly downregulated autophagy expression. Our data showed the association between autophagy and IL-1β in which reduction of autophagy promoted more IL-1β secretion and revealed that *Acanthamoeba* components modulate autophagy and IL-1β secretion differently, depending on whether macrophages are treated with *Acanthamoeba* CS or CL and whether the strain is pathogenic or nonpathogenic *Acanthamoeba*. Specifically, *Acanthamoeba* CL from both ATCC50739 and ATCC30010 induced autophagy, but only ATCC30010 CL significantly increased IL-1β secretion. In contrast, *Acanthamoeba* CS from both strains downregulated autophagy, yet only ATCC50739 CS significantly enhanced IL-1β secretion. This suggests that autophagy modulation alone does not directly determine IL-1β secretion and that strain-specific factors influence immune responses through additional pathways such as inflammasome activation, cytokine signaling, or macrophage polarization. These findings align with previous studies, demonstrating that autophagy can act as a negative regulator of IL-1β secretion by targeting pro-IL-1β for lysosomal degradation [50]. The differential response between ATCC50739 and ATCC30010 highlights the role of pathogenicity in immune modulation, with ATCC50739 CL promoting autophagy and subsequently restricted IL-1β secretion indicating a potential immune evasion strategy, while ATCC30010 CL still induced IL-1β secretion. This mechanism indicates the different characteristics of pathogenic and nonpathogenic strains and is consistent with other pathogens that manipulate autophagy to suppress inflammasome activation and cytokine secretion [51]. Interestingly, an exception was observed in ATCC30010 CS-treated macrophages, where IL-1β secretion did not increase despite autophagy impairment, suggesting that IL-1β regulation in response to nonpathogenic *Acanthamoeba* may involve alternative pathways independent of autophagy suppression.

We further manipulated autophagy either through a nutrient-depleted condition known as starvation for 4 h to induce autophagy or inhibition of autophagy by 3MA in the presence or absence of *Acanthamoeba* components and then examined the IL-1β secretion. Our results showed that autophagy induction by starvation reduced IL-1β secretion in all tested conditions including *Acanthamoeba* treatment of CS or CL of different regimens. On the other hand, inhibition of autophagy by 3MA or in combination with LPS, a classical IL-1β activator, was shown to markedly increase IL-1β secretion. However, in the presence of *Acanthamoeba* components of pathogenic or nonpathogenic strains, the IL-1β secretion was significantly reduced. This set of data confirmed the link between autophagy and IL-1β secretion and demonstrated the capability of *Acanthamoeba* components of both CS and CL to inhibit IL-1β secretion. Regarding our transcriptional analysis data, ATG6 was upregulated in starved conditions, which supports the autophagy induction during the 4-h starvation. In *Acanthamoeba* treated alone with either CS or CL, these ATG genes were remained at the basal level along the treatment. This was different from Atg8 protein expression, a key autophagy marker, in which it was reduced upon the *Acanthamoeba* CS treatment and increased after *Acanthamoeba* CL treatment. In addition, under the starvation with *A. castellanii* CS showed that the ATG6 and ATG5 mRNA expressions were at the basal level, while ATG7 was slightly increased at later time points. Similar to the starvation with *A. castellanii* CL, the expression of all tested ATG genes was gradually increased along the treatment in particular ATG7 in which the data went along with the Atg8 protein expression to support autophagy induction. This may indicate a canonical or noncanonical role of Atg7 during autophagy induction in response to *Acanthamoeba* sp. as it has been shown to play a role in several intracellular pathways such as cell death, cell cycle, and protein secretion [45]. Generally, under 3MA-treated conditions, autophagy is inhibited by blocking autophagosome formation through the inhibition of the Class III PI3K complex. This complex primarily consists of Vps34, Vps15, Atg14, Atg38, and Beclin-1 [52]. As a result, the expression of Atg8 and Atg6 is subsequently reduced [53]. Thus, upon autophagy inhibition by 3MA alone in our study, all tested ATG expressions remained unchanged and were consistent along the treatment which indicates the effectiveness of the autophagy inhibitor. In 3MA with *A. castellanii* CS- or CL-treated condition, their mRNA expression was slightly changed and finally remained at the basal level. These data indicate that *A. castellanii* CL strongly affected Atg8 expression but not other tested ATG genes, particularly in 3MA-treated condition, and those ATG expressions were completely at the basal level. Studying mRNA levels provides valuable insights into the regulation of IL-1β production, with ATG5 and ATG7 playing critical roles in modulating the autophagic degradation of pro-IL-1β and inflammasome components. These processes occur at both transcriptional and post-transcriptional levels [50], highlighting the importance of mRNA-level analyses in understanding the interplay between autophagy and inflammatory pathways. Transcriptional data also offer a snapshot of gene activation, which is essential for understanding the upstream regulation of autophagy. Notably, early autophagy induction (4–6 h) involves post-translational modifications, such as LC3 lipidation, while transcriptional regulation of genes such as ATG5, ATG6, and ATG7 becomes more evident between 6 and 12 h [54–56]. Taken together, these complementary roles of mRNA and protein-level studies provide a more comprehensive understanding of autophagy dynamics and its regulatory mechanisms.

Besides autophagy, we also investigated the transcriptional expression of inflammasome-related genes, i.e., NLRP3 and CASPASE1, including IL-1β. Our data demonstrated that in LPS- or 3MA-treated conditions, the increase in IL-1β mRNA expression was clearly observed. As expected, in *A. castellanii* 50739 CS- or CL-treated condition, IL-1β expression was significantly increased at 24 h post-treatment. However, the increased expression of NLRP3 and CASPASE1 was observed only in *Acanthamoeba* 50739 CS-treated condition. Regarding the temporal dynamics of NLRP3 expression, it is regulated by complex signaling pathways [22, 23]. The lack of a significant increase at the 4- and 24-h time points suggests that these dynamics may influence NLRP3 activation in THP-1 macrophages. Previous studies have indicated that NLRP3 often shows an initial upregulation during early time points (e.g., 6–12 h), typically peaking around 12 h post-treatment. This peak is often followed by a plateau or downregulation, likely due to negative feedback mechanisms involved in inflammasome activation [57, 58]. However, CASPASE1 may be involved in IL-1β secretion, but its expression was not markedly increased, potentially due to its constitutive presence in the cell [59, 60] or its expression following a temporal dynamic similar to NLRP3. This constitutive expression allows for a rapid host defense response by ensuring the protein is readily available for inflammasome activation without requiring significant transcriptional upregulation. Thus, its mRNA expression did not exhibit substantial changes after activation by *Acanthamoeba* treatment. This evidence further indicates that some of the IL-1β secretion mechanisms between *Acanthamoeba* CS and CL treatments might be different and in the *Acanthamoeba* CL-treated condition may be involved with NLRP3- and/or CASPASE1-independent pathway.

Ultrastructural changes of human macrophage after being exposed to the pathogenic strain of *A. castellanii* CS were observed by TEM. The reduction of pseudopodia was clearly seen. The destruction of pseudopodia might result from *Acanthamoeba* proteases, which is known as virulence factors that contribute to *Acanthamoeba* infections in human hosts [61]. Other cell morphology and organelles, such as irregular nucleus shape, increased granularity, and the presence of intracellular compartments, i.e., mitochondria, ER, a single-membrane vesicle/vacuole, and a double-membrane autophagosome, were similar to untreated control. These hallmarks of human macrophage by TEM were similar to other studies [62, 63]. Lysosomes are small, organelle-like vesicles surrounded by a single membrane [64]. However, the lysosomal structure was not characterized in our current study. Additional techniques may be needed to confirm the presence of this organelle. Nonetheless, based on our TEM data, we are confident that some of the observed vesicles/vacuoles are lysosomes. Exposure of human macrophages to *A. castellanii* CS induced IL-1β secretion. Our TEM micrographs showing small size and number of vesicles/vacuoles might be an evidence supporting the reduced IL-1β secretion following the pathogenic *A. castellanii* CS activation [65]. However, the mechanisms behind the low induction of IL-1β secretion require further investigations. In 3MA-treated macrophage which demonstrated a high IL-1β secretion, the pseudopodia were not affected upon the treatment. However, some structures were slightly changed compared to untreated control, i.e., increased autophagosome number and their accumulation at the edge of the cell as well as the presence of a large vacuole. It is possible that IL-1β is recruited to these double-membrane autophagosomes and vacuoles may be involved in the transportation of IL-1β out of the cell when autophagy pathway is inhibited [50]. While 3MA is known to inhibit canonical autophagy by blocking autophagosome formation through the suppression of Class III PI3K activity, alternative intracellular pathways may compensate for this inhibition, particularly in the context of IL-1β secretion. These mechanisms could involve noncanonical autophagy, where Atg processes independent of the canonical pathway contribute to vesicular structure formation [66]. Additionally, certain Atg proteins, such as ATG5 and ATG7, can function independently of canonical autophagy, playing critical roles in secretory autophagy, which facilitates the secretion of IL-1β and other inflammatory mediators [48]. Furthermore, the vesicular structures observed under 3MA treatment may represent alternative vesicular compartments formed by noncanonical autophagy or unrelated vesicle trafficking processes, which could also contribute to IL-1β secretion. Altogether, these compensatory or noncanonical pathways underscore the complexity of intracellular vesicular dynamics and their role in immune regulation. Interestingly, in the presence of 3MA with *A. castellanii* CS which showed the reduction of IL-1β secretion compared to 3MA-treated alone, there was also a reduction of pseudopodia number. In addition, autophagosome number was also reduced to the basal level and other structures were similar to the *A. castellanii* CS-treated alone. This indicates the ability of the secreted *Acanthamoeba* proteins in the manipulation of human macrophage and subsequently leads to the attenuation of IL-1β secretion.

Moreover, analysis of IL-1β and Atg8/LC3 protein expression and localization in human macrophage after being exposed to the pathogenic strain of *A. castellanii* CS was performed through IFA and visualized by confocal microscope. Due to the low IL-1β expression, it was unable to clearly see the IL-1β protein by confocal microscope. However, after performing image analysis by drawing the line across the representative cells in order to detect the green fluorescence signal of IL-1β, as expected, the highest green fluorescence intensity was obtained in 3MA-treated cells, and the signal was declined in the presence of *A. castellanii* CS. In untreated cells, Atg8/LC3 protein expression was clearly visible, forming puncta and localizing in the cytosol. However, in the presence of the autophagy inhibitor, 3MA, there was a reduction of Atg8/LC3 protein expression. Similarly, *A. castellanii* CS-treated cells showed partial inhibition of autophagy. Additionally, our data indicate a relationship between Atg8/LC3 and IL-1β, where an increase in the autophagy protein Atg8/LC3 leads to a decrease in IL-1β, and conversely, inhibition of autophagy results in the upregulation of IL-1β in human macrophage.

To provide a comprehensive understanding of *Acanthamoeba* pathogenesis and its impact on autophagy, this section examines both *Acanthamoeba*-secreted proteins and CL proteins, highlighting their respective roles and effects on host cellular processes. *Acanthamoeba*-secreted proteins, found in the CS, play pivotal roles in adhesion, immune modulation, and nutrient acquisition adhesion proteins such as adhesin A, *Acanthamoeba* lectin, and *Acanthamoeba* surface protein 1 enable adherence to host tissues, facilitating invasion, especially in corneal infections that can lead to severe keratitis [2]. Proteolytic enzymes, including serine proteases, cysteine proteases, metalloproteinases, and cathepsin-L, degrade host extracellular matrix components and modulate immune responses [67]. Secreted immune modulators, for example, phospholipases and heat shock proteins (HSPs), disrupt host defenses, while nutrient acquisition proteins such as siderophores and chitinases scavenge essential resources. Stress response proteins, including HSP70 and glutathione S-transferases, further enhance *Acanthamoeba*'s resilience against environmental and host-induced stress [68]. *Acanthamoeba* CL proteins, released during cell lysis, primarily support metabolic processes, oxidative stress mitigation, and nutrient acquisition. Proteins such as glutathione S-transferases and peroxiredoxins protect cellular integrity under stress [68]. Enzymes such as chitinases facilitate polysaccharide degradation, aiding survival in nutrient-limited environments [69]. Additionally, *Acanthamoeba* CL proteins may interact with host cells through DAMPs that activate TLRs, influencing immune responses [70]. The proteins derived from *Acanthamoeba* CS and CL differ significantly in their interactions with the host and their functional contributions.

The interaction between *Acanthamoeba* and macrophages is complex, as the amoeba employs various mechanisms to manipulate host immune responses. Our findings indicate that *Acanthamoeba* CL induces autophagy in macrophages. This subsequently downregulates CASPASE1 expression together with the moderate fluctuation of NLRP3 expression. This suggests that the lysate proteins may be modulating pathways in a way that supports unconventional secretion pathways for IL-1β. This is particularly interesting given that IL-1β expression and secretion were significantly increased in this context, which indicates that *Acanthamoeba* may induce IL-1β secretion via mechanisms outside the canonical inflammasome activation pathway. Certain *Acanthamoeba* CL proteins, such as those involved in the interaction with host cell machinery, may play a role in this modulation, for instance, *Acanthamoeba* HSPs [71], mannose-binding protein [72], etc., known for its involvement in disrupting host immune cell response. The interaction could further facilitate the unconventional secretion of IL-1β by altering normal cellular pathways. Conversely, *Acanthamoeba*-secreted proteins appear to reduce autophagy in macrophages, as evidenced by Atg8 protein expression. This leads to the upregulation of NLRP3 and CASPASE1 pathways, which align with the conventional mechanisms of IL-1β secretion. This suggests that specific *Acanthamoeba* proteins might inhibit autophagic processes, thereby promoting a classical inflammasome-mediated response. For instance, the *Acanthamoeba* cysteine proteases [24], phospholipases [73], and *Acanthamoeba*-derived exosomes contain various proteins and microRNAs [74] that could elicit Atg responses in macrophages, thus emphasizing the potential of these secreted proteins in orchestrating immune responses. Such modulation can further lead to enhanced IL-1β expression and secretion through the conventional pathway, underscoring the dual strategies employed by *Acanthamoeba* to manipulate macrophage responses. Collectively, *Acanthamoeba* possesses sophisticated mechanisms to influence autophagy in macrophages, with its proteins either promoting or inhibiting this pathway, thereby altering the inflammatory milieu. Further exploration into the specific roles and mechanisms of these *Acanthamoeba*-derived proteins will deepen our understanding of host–pathogen interactions and may reveal novel therapeutic targets for diseases associated with *Acanthamoeba* infections.

Regarding the role of autophagy in the regulation of inflammasome and subsequently control IL-1β secretion [22, 28], inflammasome is a multiprotein complex responsible for IL-1β processing before secretion [23]. Upon cell activation, pro-IL-1β is cleaved by the inflammasome containing caspase-1, and then, the mature form of IL-1β is secreted out of the cell [10, 23]. Autophagy induction degrades inflammasome components resulting in a reduction of IL-1β secretion [22, 75]. This evidence further indicates a regulation of inflammatory response to infectious diseases [20]. To our data, the possible mechanisms of IL-1β secretion from surviving human macrophages upon *Acanthamoeba* component activation are described herein: (i) *Microvesicle shedding*, which is an early and rapid mechanism for IL-1β secretion from activated immune cells [76]; (ii) *IL-1*β*-containing vesicles/lysosomes* in which the IL-1β is recruited into vesicles/lysosomes. Then, the IL-1β-containing vesicles/lysosomes can be further targeted for degradation or rescued for IL-1β secretion called lysosome exocytosis which depends on the stimuli [65]; (iii) *Lysosome exocytosis during pyroptosis*, pyroptosis is a regulated form of cell death that relies on caspase-1 activation. It is characterized by the rapid rupture of the plasma membrane, releasing proinflammatory molecules through caspase-1-dependent pore formation. This process also requires the participation of NLRP3 and gasdermin D (GSDMD) to induce pyroptosis-mediated cytokine release and ultimately lead to osmotic cell lysis, known as a canonical inflammasome [77]. However, this mechanism is suggested as a cell survival mechanism for repairing cell membrane rather than as a mechanism of cytokine secretion [78], and in our study, the dose of *Acanthamoeba* components used did not induce cell death, as confirmed by the MTT assay as well as the TEM micrographs also showed no signs of nuclear condensation, cell swelling, or membrane rupture. Therefore, cell death mechanisms such as apoptosis and pyroptosis were excluded [79]; (iv) *Autophagy*, IL-1β is recruited to a double-membrane autophagosome. Thus, the IL-1β-containing autophagosome is further targeted for degradation or secretion depending on the autophagy activation or inhibition, respectively [50]. Although our data show that macrophage autophagy can be either increased or decreased depending on the type of *Acanthamoeba* component activation, basal autophagy remains active and likely promotes IL-1β secretion; (v) *Chaperone (HSP90)-mediated IL-1β translocation into LC3-positive vesicles*, a vesicle, potentially a precursor of the phagophore, contains translocated mature IL-1β and subsequently develops into an autophagosome, with the mature IL-1β located within the intermembrane space of the double-membrane structure. The completion of IL-1β secretion involves the Golgi reassembly and stacking proteins (GRASPs) and the formation of multivesicular bodies (MVBs) [80]. This mechanism involves autophagy and is potentially related to IL-1β secretion, particularly under *Acanthamoeba* CL treatment, which has been shown to induce autophagy in macrophages; (vi) *Lysosomal damage and secretory autophagy*, IL-1β is identified by the specialized secretory autophagy cargo receptor TRIM16, which interacts with the R‐SNARE Sec22b to direct cargo to LC3-II-positive membranes. At the plasma membrane, the protein complex further allows IL-1β exocytosis and secretion. Additionally, Sec22b, previously noted for its ability to tether membranes to the plasma membrane without fusion, may position and concentrate TRIM16-mIL-1β complexes at the plasma membrane for secretion, translocation, or release mechanisms through pores [81]. However, further research is necessary to identify the proteins involved in the secretion process to clarify the last two secretion mechanisms. Moreover, a noncanonical inflammasome might be involved in IL-1β secretion [82] as the NLRP3 and CASPASE1 were not upregulated following *Acanthamoeba* treatment.

IL-1β is a key proinflammatory cytokine involved in host defense against parasitic infections, primarily by recruiting neutrophils and macrophages, enhancing phagocytosis, and regulating immune responses [17, 28]. In acute parasitic infections, IL-1β typically acts as an early immune responder, triggering inflammasome activation and amplifying cytokine signaling. For example, in *Toxoplasma gondii* [42] and *Plasmodium* spp. [83] infections, IL-1β is rapidly released through NLRP3 inflammasome activation, promoting parasite clearance. However, some parasites, such as *Leishmania donovani*, suppress IL-1β production to evade immune detection and facilitate intracellular survival [84]. Similarly, our study suggests that *Acanthamoeba* infection results in remarkably low IL-1β secretion, potentially due to its ability to modulate macrophage autophagy, which in turn prevents efficient inflammasome activation and cytokine release. This suppression may impair early immune responses, delaying pathogen clearance and promoting prolonged infection. In chronic parasitic infections, sustained IL-1β secretion can contribute to excessive inflammation and tissue damage. For example, prolonged IL-1β signaling in *Leishmania major* infection exacerbates chronic inflammation, leading to granuloma formation [85]. In *Acanthamoeba* infections, despite multiple potential sources of IL-1β including neutrophils, dendritic cells, and epithelial cells, the overall secretion remains low [2]. This may allow *Acanthamoeba* to persist within host tissues and contribute to prolonged infections such as chronic AK and GAE. Additionally, while our in vitro findings provide valuable insights into *Acanthamoeba*-driven immune modulation, the in vivo environment is likely more complex, where macrophages interact with cytokine networks, tissue-resident immune cells, and disseminating pathogens, potentially leading to distinct immune responses. The extent to which *Acanthamoeba* CS and CL regulate autophagy and IL-1β secretion may vary based on tissue localization and infection stage [86]. Thus, further in vivo studies are needed to assess the dynamic interplay between *Acanthamoeba* spp., autophagy, and IL-1β secretion in different infection models. Taken together, our findings suggest that *Acanthamoeba* employs an immune evasion strategy by modulating autophagy in macrophages to control IL-1β secretion, potentially facilitating pathogen persistence. In addition, the differential regulation of autophagy- and inflammasome-related pathways highlights multiple mechanisms involved in IL-1β secretion during *Acanthamoeba* infection.

## 5. Conclusions

Our study highlights the ability of *Acanthamoeba* components to regulate autophagy and subsequently control IL-1β secretion at low levels in human macrophages, indicating an immune evasion mechanism by the parasite. The differing transcriptional expression patterns of autophagy- and inflammasome-related genes in response to *Acanthamoeba* CS and CL suggest that *Acanthamoeba* employs multiple pathways to regulate UPS of IL-1β. This regulation may allow *Acanthamoeba* to manipulate macrophage responses, dampening inflammation and facilitating pathogen persistence within host tissues. Further characterization of Atg proteins involved in cytokine secretion and autophagy-associated pathways, such as the proteasome, along with an exploration of macrophage functions, including intracellular defense mechanisms, could enhance our understanding of IL-1β secretion and its regulation via autophagy. To better understand immune responses in *Acanthamoeba* infections, in vivo studies on different *Acanthamoeba* species and macrophage phenotypes during early and late infection are needed to clarify inflammatory regulation and immune cell coordination. Investigating chronic infection models will help determine the long-term role of macrophages and IL-1β in immune regulation, parasite persistence, and tissue pathology, providing new insights into chronic *Acanthamoeba* infections. Additionally, profiling the proteins secreted by *Acanthamoeba* in the CS of both pathogenic and nonpathogenic strains could provide further insight into the indirect activation of immune cells by microbial components. A comprehensive understanding of these mechanisms could ultimately lead to innovative therapeutic strategies targeting autophagy modulation or macrophage responses to better control *Acanthamoeba* infections.

## Data Availability

Data are available in the article supporting information.

## Supporting Information

Figure S1 IL-1β secretion in response to *Acanthamoeba castellanii* culture supernatant and cell lysate. THP-1 macrophages were treated with *A*. *castellanii* ATCC50739 (A, B), *A. castellanii* ATCC30010 (C, D), and *Acanthamoeba* culture medium, PYG, alone (E). Different amount (%v/v) of *Acanthamoeba* CS obtained from Days 1, 3, and 5 were tested as well as *Acanthamoeba* CL at 10% (18 μg/mL). LPS-treated condition was used as a positive control for IL-1β secretion. Dotted line represented a basal level of IL-1β in THP-1 macrophage culture, 16.40 pg/mL (Untreated cells). Data are obtained from three independent experiments and represented as mean ± SD. ⁣^∗^*p* < 0.05; ⁣^∗∗^*p* < 0.01; ⁣^∗∗∗^*p* < 0.001.

Figure S2 Western blot analysis of LC3 and IL-1β expression. (A) THP-1 macrophages were starved for 4 h and treated with 3MA (1 mM) or LPS (1 μg/mL) for 24 h. Untreated THP-1 monocyte and macrophage were included. Cells were harvested for immunoblotting and probed with anti-LC3. (B) THP-1 macrophages were starved or treated with 3MA in the presence or absence of *A. castellanii* ATCC50739 culture supernatant (CS) or cell lysate (CL). Cells were harvested at 24 h post-treatment and probed with anti-IL-1β after the membrane blotting. Anti-actin was included as a control. The molecular weight of target proteins in kDa was shown on the left.

Figure S3 Cytotoxicity analysis by MTT assay. (A) Different conditions of *A. castellanii* culture supernatant (CS) and cell lysate (CL)-treated THP-1 macrophages were examined for cell viability at 24 h post-treatment by MTT assay. *A. castellanii* CS harvested from Days 1, 3, and 5 was used for THP-1 treatment at 2%, 6%, and 10% while *A. castellanii* CL harvested from Day 3 was used for THP-1 treatment at 4, 8, and 16 μg/mL. (B) THP-1 macrophage-treated conditions under autophagy manipulation were also tested for their cell viability. %cell viability was calculated. Bar graphs represent mean ± SD. Data are obtained from three independent experiments.

Figure S4 Quantification of LC3 puncta per cell in THP-1 macrophages. The cells were pulsed with *A. castellanii* ATCC50739 culture supernatant, 3MA, or the combination of the culture supernatant and 3MA. Untreated condition was included as a control. The immunofluorescence assay was performed to observe the expression of human Atg8/LC3. The LC3 puncta-containing cells were quantified by analyzing at least 100 cells per condition from two independent experiments. Only puncta ≥ 0.25 μm in size were counted. Bar graphs represent mean ± SD. ⁣^∗∗∗^*p* < 0.001.

## References

[1] Bunsuwansakul C., Mahboob T., Hounkong K. (2019). *Acanthamoeba* in Southeast Asia–Overview and Challenges. *Korean Journal of Parasitology*.

[2] Khan N. A. (2006). *Acanthamoeba*: Biology and Increasing Importance in Human Health. *FEMS Microbiology Reviews*.

[3] Khan N. A., Anwar A., Siddiqui R. (2019). *Acanthamoeba* Keratitis: Current Status and Urgent Research Priorities. *Current Medicinal Chemistry*.

[4] Lorenzo-Morales J., Martín-Navarro C. M., López-Arencibia A., Arnalich-Montiel F., Piñero J. E., Valladares B. (2013). *Acanthamoeba* Keratitis: An Emerging Disease Gathering Importance Worldwide?. *Trends in Parasitology*.

[5] Clarke D. W., Niederkorn J. Y. (2006). The Immunobiology of *Acanthamoeba* Keratitis. *Microbes and Infection*.

[6] Niederkorn J. Y. (2002). The Role of the Innate and Adaptive Immune Responses in *Acanthamoeba* Keratitis. *Archivum Immunologiae et Therapiae Experimentalis*.

[7] Kot K., Łanocha-Arendarczyk N., Kosik-Bogacka D. (2021). Immunopathogenicity of *Acanthamoeba* spp. in the Brain and Lungs. *International Journal of Molecular Sciences*.

[8] Quan J.-H., Huang R., Wang Z. (2018). P2X7 Receptor Mediates NLRP3-Dependent IL-1β Secretion and Parasite Proliferation in *Toxoplasma gondii*-Infected Human Small Intestinal Epithelial Cells. *Parasites & Vectors*.

[9] Voronov E., Dotan S., Gayvoronsky L. (2010). IL-1-Induced Inflammation Promotes Development of Leishmaniasis in Susceptible BALB/c Mice. *International Immunology*.

[10] Lopez-Castejon G., Brough D. (2011). Understanding the Mechanism of IL-1β Secretion. *Cytokine & Growth Factor Reviews*.

[11] Dinarello C. A. (2018). Overview of the IL‐1 Family in Innate Inflammation and Acquired Immunity. *Immunological Reviews*.

[12] Voronov E., Dotan S., Krelin Y. (2013). Unique Versus Redundant Functions of IL-1α and IL-1β in the Tumor Microenvironment. *Frontiers in Immunology*.

[13] Takeuchi O., Akira S. (2010). Pattern Recognition Receptors and Inflammation. *Cell*.

[14] Cohen P. (2014). The TLR and IL-1 Signalling Network at a Glance. *Journal of Cell Science*.

[15] Rabouille C., Malhotra V., Nickel W. (2012). Diversity in Unconventional Protein Secretion. *Journal of Cell Science*.

[16] Deretic V., Levine B. (2009). Autophagy, Immunity, and Microbial Adaptations. *Cell Host & Microbe*.

[17] Starling S. (2018). Innate Immunity: Revealing the Secrets of IL-1 Secretion. *Nature Reviews Immunology*.

[18] Claude-Taupin A., Bissa B., Jia J., Gu Y., Deretic V. (2018). Role of Autophagy in IL-1β Export and Release from Cells. *Seminars in Cell & Developmental Biology*.

[19] Palomo J., Dietrich D., Martin P., Palmer G., Gabay C. (2015). The Interleukin (IL)-1 Cytokine Family–Balance between Agonists and Antagonists in Inflammatory Diseases. *Cytokine*.

[20] Ying Y., Sun C.-B., Zhang S.-Q. (2021). Induction of Autophagy via the TLR4/NF-Κb Signaling Pathway by Astragaloside IV Contributes to the Amelioration of Inflammation in RAW264. 7 Cells. *Biomedicine & Pharmacotherapy*.

[21] Kimura T., Jia J., Claude-Taupin A. (2017). Cellular and Molecular Mechanism for Secretory Autophagy. *Autophagy*.

[22] Biasizzo M., Kopitar-Jerala N. (2020). Interplay Between NLRP3 Inflammasome and Autophagy. *Frontiers in Immunology*.

[23] Kelley N., Jeltema D., Duan Y., He Y. (2019). The NLRP3 Inflammasome: An Overview of Mechanisms of Activation and Regulation. *International Journal of Molecular Sciences*.

[24] Cano A., Mattana A., Henriquez F. L., Alexander J., Roberts C. W. (2019). Acanthamoeba Proteases Contribute to Macrophage Activation Through PAR1, but Not PAR2. *Parasite Immunology*.

[25] Beltan E., Horgen L., Rastogi N. (2000). Secretion of Cytokines by Human Macrophages Upon Infection by Pathogenic and Non-Pathogenic Mycobacteria. *Microbial Pathogenesis*.

[26] Kim Y.-M., Talanian R. V., Li J., Billiar T. R. (1998). Nitric Oxide Prevents IL-1β and IFN-γ-Inducing Factor (IL-18) Release From Macrophages by Inhibiting Caspase-1 (IL-1β-Converting Enzyme). *The Journal of Immunology*.

[27] Rolot M., Dewals B. G. (2018). Macrophage Activation and Functions during Helminth Infection: Recent Advances From the Laboratory Mouse. *Journal of immunology research*.

[28] Netea M. G., Simon A., van de Veerdonk F., Kullberg B.-J., Van der Meer J. W., Joosten L. A. (2010). IL-1β Processing in Host Defense: Beyond the Inflammasomes. *PLoS Pathogens*.

[29] Peiró T., Patel D. F., Akthar S. (2018). Neutrophils Drive Alveolar Macrophage IL-1β Release During Respiratory Viral Infection. *Thorax*.

[30] Taravaud A., Loiseau P. M., Pomel S. (2017). *In Vitro* Evaluation of Antimicrobial Agents on *Acanthamoeba* sp. and Evidence of a Natural Resilience to Amphotericin B. *International Journal for Parasitology: Drugs and Drug Resistance*.

[31] Giambelluca S., Ochs M., Lopez-Rodriguez E. (2022). Resting Time After Phorbol 12-Myristate 13-Acetate in THP-1 Derived Macrophages Provides a Non-Biased Model for the Study of NLRP3 Inflammasome. *Frontiers in Immunology*.

[32] Hiransai P., Tangpong J., Kumbuar C. (2016). Anti-Nitric Oxide Production, Anti-Proliferation and Antioxidant Effects of the Aqueous Extract From Tithonia Diversifolia. *Asian Pacific Journal of Tropical Biomedicine*.

[33] Deng Q., Wang Z., Wang L. (2013). Lower mRNA and Protein Expression Levels of LC3 and Beclin1, Markers of Autophagy, Were Correlated With Progression of Renal Clear Cell Carcinoma. *Japanese Journal of Clinical Oncology*.

[34] Yi K., Yang Y., Yuan Y., Xiang Y., Zhou S. (2022). Impaired Autophagy Causes Severe Corneal Neovascularization. *Cells*.

[35] Rouschop K. M., van den Beucken T., Dubois L. (2010). The Unfolded Protein Response Protects Human Tumor Cells During Hypoxia Through Regulation of the Autophagy Genes MAP1LC3B and ATG5. *Journal of Clinical Investigation*.

[36] Jiang W., Lv H., Wang H. (2015). Activation of the NLRP3/Caspase-1 Inflammasome in Human Dental Pulp Tissue and Human Dental Pulp Fibroblasts. *Cell and Tissue Research*.

[37] Han C., Wang W. (2018). MicroRNA-129-5p Suppresses Cell Proliferation, Migration and Invasion via Targeting ROCK1 in Osteosarcoma. *Molecular Medicine Reports*.

[38] Boonhok R., Sangkanu S., Phumjan S. (2022). Curcumin Effect on Acanthamoeba Triangularis Encystation Under Nutrient Starvation. *PeerJ*.

[39] Baral P., Utaisincharoen P. (2013). Sterile-α-and Armadillo Motif-Containing Protein Inhibits the TRIF-Dependent Downregulation of Signal Regulatory Protein α to Interfere With Intracellular Bacterial Elimination in *Burkholderia pseudomallei*-Infected Mouse Macrophages. *Infection and Immunity*.

[40] Boonhok R., Rachaphaew N., Duangmanee A. LAP-Like Process as an Immune Mechanism Downstream of IFN-γ in Control of the Human Malaria Plasmodium Vivax Liver Stage.

[41] Patil T., More V., Rane D. (2018). Pro-Inflammatory Cytokine Interleukin-1β (IL-1β) Controls Leishmania Infection. *Cytokine*.

[42] Pandori W. J., Matsuno S. Y., Shin J.-H. (2024). Role for Caspase-8 in the Release of IL-1β and Active Caspase-1 From Viable Human Monocytes During *Toxoplasma gondii* Infection. *The Journal of Immunology*.

[43] Ponpuak M., Mandell M. A., Kimura T., Chauhan S., Cleyrat C., Deretic V. (2015). Secretory Autophagy. *Current Opinion in Cell Biology*.

[44] Martens S., Fracchiolla D. (2020). Activation and Targeting of ATG8 Protein Lipidation. *Cell discovery*.

[45] Collier J., Suomi F., Olahova M., McWilliams T., Taylor R. (2021). Emerging Roles of ATG7 in Human Health and Disease. *EMBO Molecular Medicine*.

[46] Pu Q., Gan C., Li R. (2017). Atg7 Deficiency Intensifies Inflammasome Activation and Pyroptosis in *Pseudomonas* Sepsis. *The Journal of Immunology*.

[47] Walczak M., Martens S. (2013). Dissecting the Role of the Atg12–Atg5-Atg16 Complex During Autophagosome Formation. *Autophagy*.

[48] Dupont N., Jiang S., Pilli M., Ornatowski W., Bhattacharya D., Deretic V. (2011). Autophagy-Based Unconventional Secretory Pathway for Extracellular Delivery of IL-1β: Autophagy-Based Unconventional Secretory Pathway. *The EMBO Journal*.

[49] Kang R., Zeh H., Lotze M., Tang D. (2011). The Beclin 1 Network Regulates Autophagy and Apoptosis. *Cell Death & Differentiation*.

[50] Harris J., Hartman M., Roche C. (2011). Autophagy Controls IL-1β Secretion by Targeting Pro-IL-1β for Degradation. *Journal of Biological Chemistry*.

[51] Seveau S., Turner J., Gavrilin M. A. (2018). Checks and Balances Between Autophagy and Inflammasomes During Infection. *Journal of Molecular Biology*.

[52] Nemazanyy I., Montagnac G., Russell R. C. (2015). Class III PI3K Regulates Organismal Glucose Homeostasis by Providing Negative Feedback on Hepatic Insulin Signalling. *Nature Communications*.

[53] Wei X., Zhou Z., Li L. (2016). Intrathecal Injection of 3-Methyladenine Reduces Neuronal Damage and Promotes Functional Recovery via Autophagy Attenuation After Spinal Cord Ischemia/Reperfusion Injury in Rats. *Biological and Pharmaceutical Bulletin*.

[54] Bellot G., Garcia-Medina R., Gounon P. (2009). Hypoxia-Induced Autophagy Is Mediated through Hypoxia-Inducible Factor Induction of BNIP3 and BNIP3L via Their BH3 Domains. *Molecular and Cellular Biology*.

[55] Mizushima N., Komatsu M. (2011). Autophagy: Renovation of Cells and Tissues. *Cell*.

[56] Ogata M., Hino S.-I., Saito A. (2006). Autophagy Is Activated for Cell Survival After Endoplasmic ReticulumStress. *Molecular and Cellular Biology*.

[57] Awad F., Assrawi E., Jumeau C. (2017). Impact of Human Monocyte and Macrophage Polarization on NLR Expression and NLRP3 Inflammasome Activation. *PLoS One*.

[58] Zhao W., Ma L., Cai C., Gong X. (2019). Caffeine Inhibits NLRP3 Inflammasome Activation by Suppressing MAPK/NF-κB and A2aR Signaling in LPS-Induced THP-1 Macrophages. *International Journal of Biological Sciences*.

[59] Broz P., Dixit V. M. (2016). Inflammasomes: Mechanism of Assembly, Regulation and Signalling. *Nature Reviews Immunology*.

[60] Dinarello C. A. (2009). Immunological and Inflammatory Functions of the Interleukin-1 Family. *Annual Review of Immunology*.

[61] de Souza Carvalho F., Carrijo-Carvalho L., Chudzinski-Tavassi A. M., Foronda A. S., De Freitas D. (2011). Serine-Like Proteolytic Enzymes Correlated With Differential Pathogenicity in Patients With Acute *Acanthamoeba* Keratitis. *Clinical Microbiology and Infection*.

[62] Gatto F., Cagliani R., Catelani T. (2017). PMA-Induced THP-1 Macrophage Differentiation Is Not Impaired by Citrate-Coated Platinum Nanoparticles. *Nanomaterials*.

[63] Sadofsky L. R., Hayman Y. A., Vance J. (2019). Characterisation of a New Human Alveolar Macrophage-Like Cell Line (Daisy). *Lung*.

[64] Winchester B. G. (2001). Lysosomal Membrane Proteins. *European Journal of Paediatric Neurology*.

[65] Andrei C., Dazzi C., Lotti L., Torrisi M. R., Chimini G., Rubartelli A. (1999). The Secretory Route of the Leaderless Protein Interleukin 1β Involves Exocytosis of Endolysosome-Related Vesicles. *Molecular Biology of the Cell*.

[66] Galluzzi L., Baehrecke E. H., Ballabio A. (2017). Molecular Definitions of Autophagy and Related Processes. *The EMBO Journal*.

[67] Betanzos A., Bañuelos C., Orozco E. (2019). Host Invasion by Pathogenic Amoebae: Epithelial Disruption by Parasite Proteins. *Genes*.

[68] Hasni I., Andréani J., Colson P., La Scola B. (2020). Description of Virulent Factors and Horizontal Gene Transfers of Keratitis-Associated Amoeba *Acanthamoeba triangularis* by Genome Analysis. *Pathogens*.

[69] Magistrado-Coxen P., Aqeel Y., Lopez A. (2019). The Most Abundant Cyst Wall Proteins of *Acanthamoeba castellanii* Are Lectins That Bind Cellulose and Localize to Distinct Structures in Developing and Mature Cyst Walls. *PLoS Neglected Tropical Diseases*.

[70] Cano A., Mattana A., Woods S., Henriquez F. L., Alexander J., Roberts C. W. (2017). *Acanthamoeba* Activates Macrophages Predominantly Through Toll-Like Receptor 4-and MyD88-Dependent Mechanisms to Induce Interleukin-12 (IL-12) and IL-6. *Infection and Immunity*.

[71] Wang N., Sun H., Liu D. (2020). Ac-HSP20 Is Associated With the Infectivity and Encystation of *Acanthamoeba castellanii*. *Frontiers in Microbiology*.

[72] Garate M., Marchant J., Cubillos I., Cao Z., Khan N. A., Panjwani N. (2006). *In Vitro* Pathogenicity of *Acanthamoeba* Is Associated With the Expression of the Mannose-Binding Protein. *Investigative Opthalmology & Visual Science*.

[73] Marciano-Cabral F., Cabral G. (2003). *Acanthamoeba* spp. as Agents of Disease in Humans. *Clinical Microbiology Reviews*.

[74] Costa A. O., Chagas I. A. R., de Menezes‐Neto A. (2021). Distinct Immunomodulatory Properties of Extracellular Vesicles Released by Different Strains of *Acanthamoeba*. *Cell Biology International*.

[75] Saitoh T., Akira S. (2016). Regulation of Inflammasomes by Autophagy. *Journal of Allergy and Clinical Immunology*.

[76] Surprenant A. (2001). Rapid Secretion of Interleukin-1beta by Microvesicle Shedding. *Immunity*.

[77] Galluzzi L., Spetz J. K. (2020). *Cell Death Regulation in Health and Disease-Part B*.

[78] Bergsbaken T., Fink S. L., Den Hartigh A. B., Loomis W. P., Cookson B. T. (2011). Coordinated Host Responses During Pyroptosis: Caspase-1–Dependent Lysosome Exocytosis and Inflammatory Cytokine Maturation. *The Journal of Immunology*.

[79] Ketelut-Carneiro N., Fitzgerald K. A. (2022). Apoptosis, Pyroptosis, and Necroptosis—Oh My! The Many Ways a Cell Can Die. *Journal of Molecular Biology*.

[80] Zhang M., Kenny S. J., Ge L., Xu K., Schekman R. (2015). Translocation of Interleukin-1β Into a Vesicle Intermediate in Autophagy-Mediated Secretion. *Elife*.

[81] Kimura T., Jia J., Kumar S. (2017). Dedicated SNARE S and Specialized TRIM Cargo Receptors Mediate Secretory Autophagy. *The EMBO Journal*.

[82] Kelly P., Meade K. G., O’Farrelly C. (2019). Non-Canonical Inflammasome-Mediated IL-1β Production by Primary Endometrial Epithelial and Stromal Fibroblast Cells Is NLRP3 and Caspase-4 Dependent. *Frontiers in Immunology*.

[83] Tiemi Shio M., Eisenbarth S. C., Savaria M. (2009). Malarial Hemozoin Activates the NLRP3 Inflammasome Through Lyn and Syk Kinases. *PLoS Pathogens*.

[84] Gupta P., Srivastav S., Saha S., Das P. K., Ukil A. (2016). Leishmania Donovani Inhibits Macrophage Apoptosis and Pro-Inflammatory Response Through AKT-Mediated Regulation of β-Catenin and FOXO-1. *Cell Death & Differentiation*.

[85] Scharton T. M., Scott P. (1993). Natural Killer Cells Are a Source of Interferon Gamma That Drives Differentiation of CD4+ T Cell Subsets and Induces Early Resistance to *Leishmania major* in Mice. *Journal of Experimental Medicine*.

[86] Marciano‐Cabral F., Toney D. M. (1998). The Interaction of *Acanthamoeba* spp. With Activated Macrophages and With Macrophage Cell Lines. *The Journal of Eukaryotic Microbiology*.

