# Supplementary material for: Acanthamoeba castellanii–Mediated Reduction of Interleukin-1β Secretion and Its Association With Macrophage Autophagy
Source: Scientifica (Cairo). 2025 Mar 12;2025:3430892. doi: 10.1155/sci5/3430892 (PMC11922611; doi:10.1155/sci5/3430892)
Supplement: Supporting Information — Additional supporting information can be found online in the Supporting Information section. [file 3430892.f1.docx]

***Acanthamoeba castellanii*-Mediated Reduction of Interleukin-1β Secretion and Its Association with Macrophage Autophagy**

Rachasak Boonhok^1^, Wilaiwan Senghoi^2^, Suthinee Sangkanu^3^, Chooi Ling Lim^4^, Matsayapan Pudla^5^, Maria de Lourdes Pereira^6^, Polrat Wilairatana^7^, Tooba Mahboob^8^, Md. Atiar Rahman^9^, Pongsak Utaisincharoen^10^, Poonsit Hiransai^11*^, Veeranoot Nissapatorn^12*^

**Supplementary data**

**
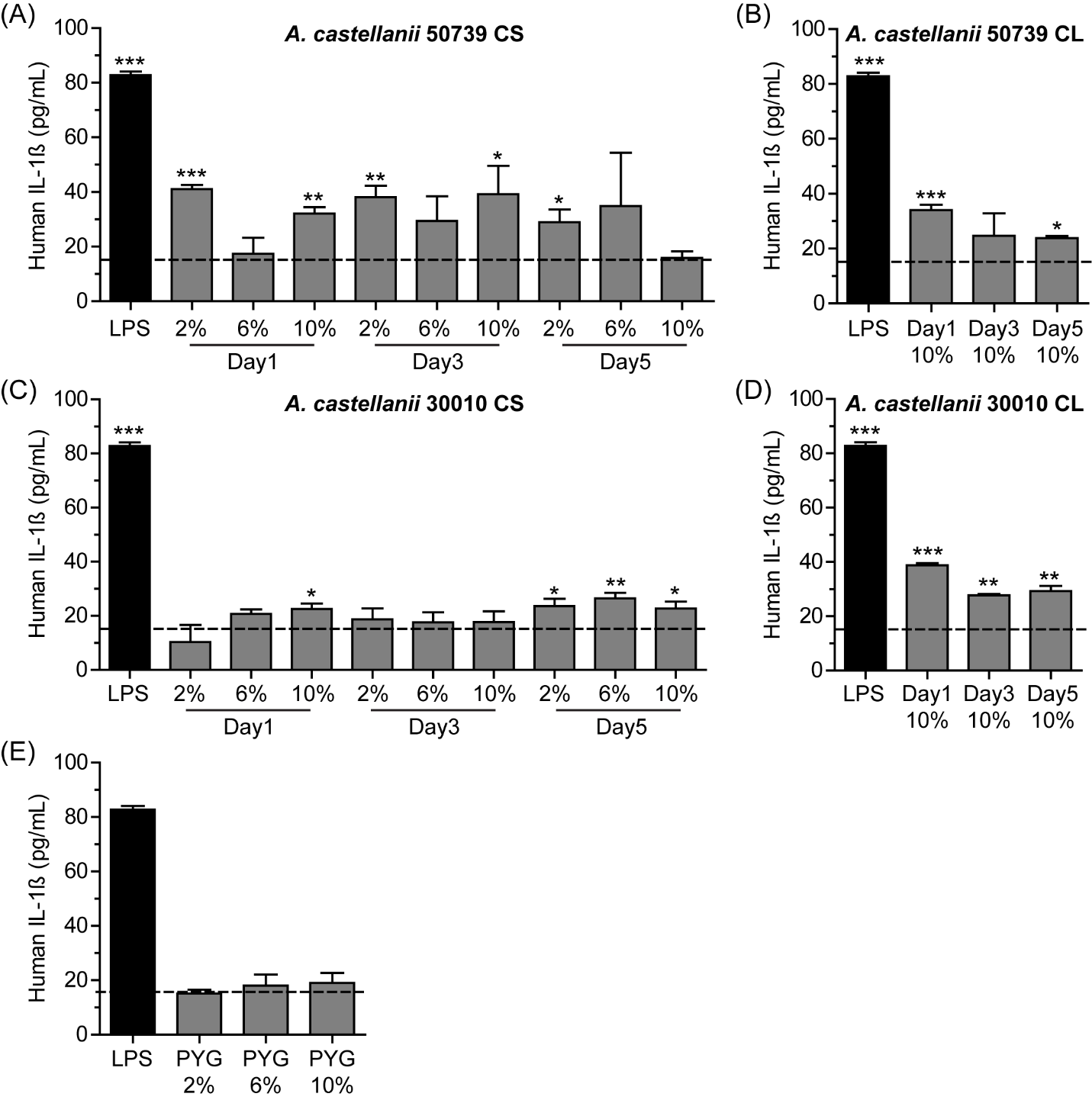
**

**Fig. S1 IL-1ß secretion in response to *Acanthamoeba castellanii* culture supernatant and cell lysate.** THP-1 macrophages were treated with *A. castellanii* ATCC50739 **(A, B)**, *A. castellanii* ATCC30010 **(C, D)**, and *Acanthamoeba* culture medium, PYG, alone **(E)**. Different amount (%v/v) of *Acanthamoeba* CS obtained from Day1, 3, 5 were tested as well as *Acanthamoeba* CL at 10% (18 µg/mL). LPS-treated condition was used as a positive control for IL-1ß secretion. Dotted line represented a basal level of IL-1ß in THP-1 macrophage culture, 16.40 pg/mL (Untreated cells). Data obtained from 3-independent experiments and represented as mean ± SD. *, *p* < 0.05; **, *p* < 0.01; ***, *p* < 0.001.


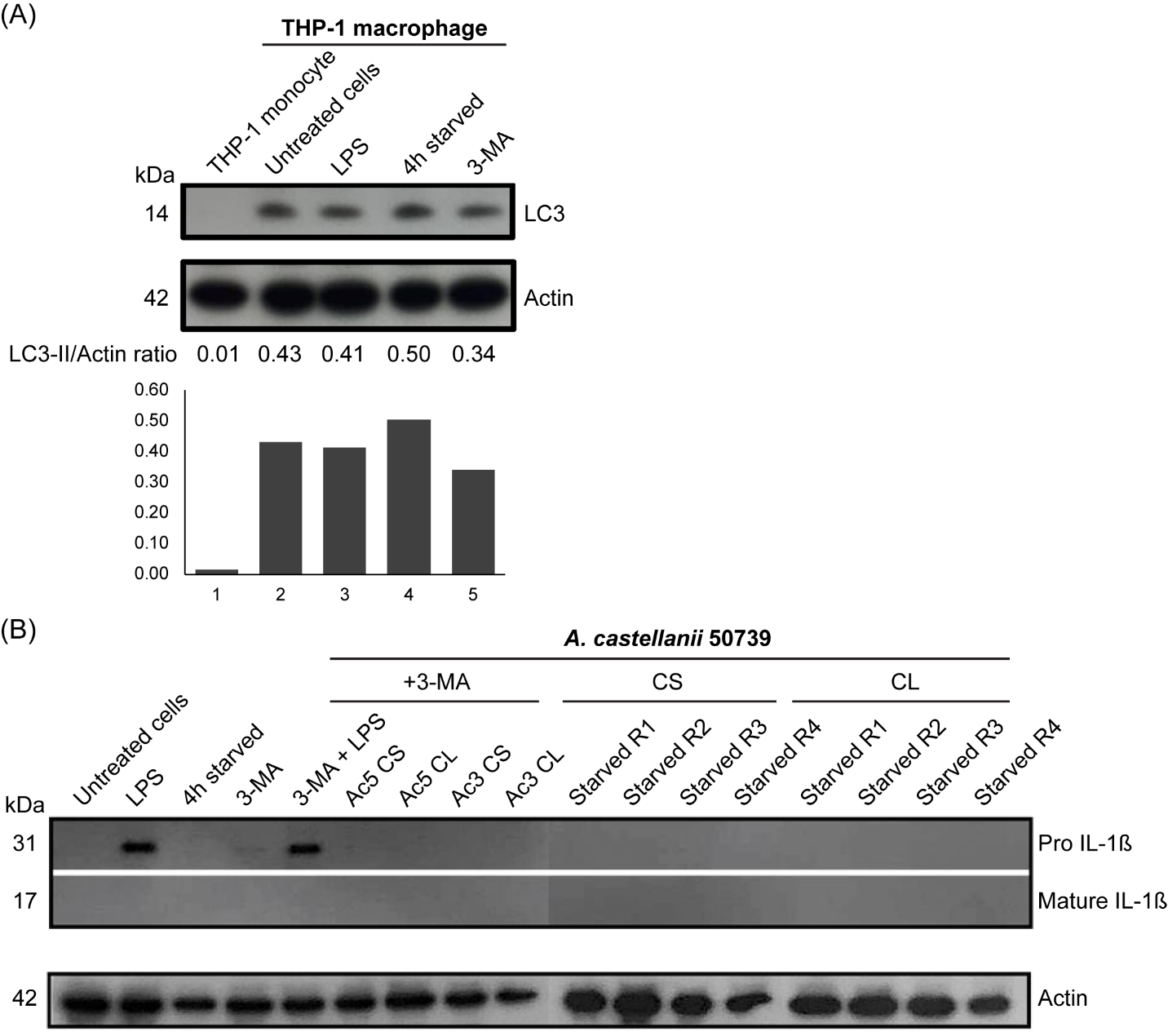


**Fig. S2** **Western blot analysis of LC3 and IL-1ß expression. (A)** THP-1 macrophages were starved for 4 h and treated with 3MA (1mM) or LPS (1 µg/mL) for 24 h. Untreated THP-1 monocyte and macrophage were included. Cells were harvested for immunoblotting and probed with anti-LC3. **(B)** THP-1 macrophages were starved or treated with 3MA in the presence or absence of *A. castellanii* ATCC50739 culture supernatant (CS) or cell lysate (CL). Cells were harvested at 24 h post treatment and probed with anti-IL-1ß after the membrane blotting. Anti-actin was included as a control. The molecular weight of target proteins in kDa was shown on the left.

**
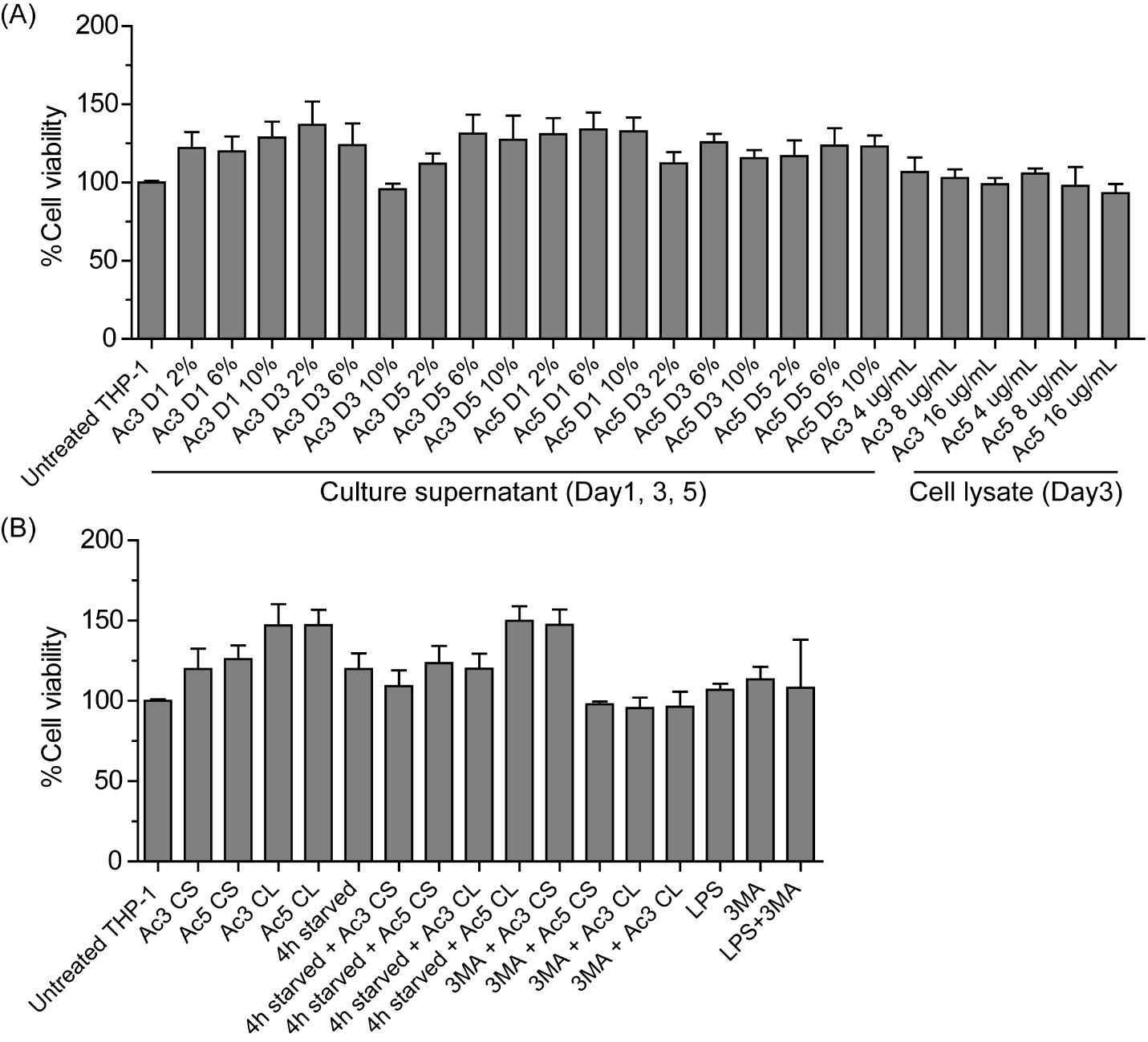
**

**Fig. S3** **Cytotoxicity analysis by MTT assay.** **(A)** Different conditions of *A. castellanii* culture supernatant (CS) and cell lysate (CL)-treated THP-1 macrophages were examined for cell viability at 24 h post treatment by MTT assay. *A. castellanii* CS harvested from Day1, 3, 5 was used for THP-1 treatment at 2%, 6%, and 10% while *A. castellanii* CL harvested from Day3 was used for THP-1 treatment at 4, 8, 16 µg/mL. **(B)** THP-1 macrophage-treated conditions under autophagy manipulation were also tested for their cell viability. %cell viability was calculated. Bar graphs represent mean ± SD. Data obtained from 3-independent experiments.


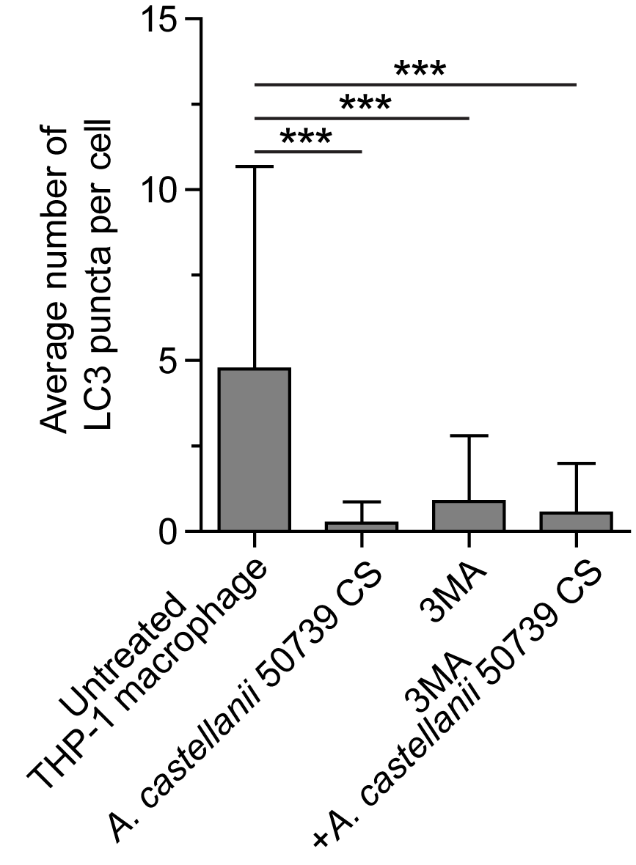


**Fig. S4** **Quantification of LC3 puncta per cell in THP-1 macrophages.** The cells were pulsed with *A. castellanii* ATCC50739 culture supernatant, 3MA, or the combination of the culture supernatant and 3MA. Untreated condition was included as a control. The immunofluorescence assay was performed to observe the expression of human Atg8/LC3. The LC3 puncta-containing cells were quantified by analyzing at least 100 cells per condition from 2-independent experiments. Only puncta ≥ 0.25 µm in size were counted. Bar graphs represent mean ± SD. ***, *p* < 0.001.
